# Lysosomal degradation of newly formed insulin granules contributes to β cell failure in diabetes

**DOI:** 10.1038/s41467-019-11170-4

**Published:** 2019-07-25

**Authors:** Adrien Pasquier, Kevin Vivot, Eric Erbs, Coralie Spiegelhalter, Zhirong Zhang, Victor Aubert, Zengzhen Liu, Meryem Senkara, Elisa Maillard, Michel Pinget, Julie Kerr-Conte, François Pattou, Gilbert Marciniak, Axel Ganzhorn, Paolo Ronchi, Nicole L. Schieber, Yannick Schwab, Paul Saftig, Alexander Goginashvili, Romeo Ricci

**Affiliations:** 1 Institut de Génétique et de Biologie Moléculaire et Cellulaire, 67404 Illkirch, France; 2 Centre National de la Recherche Scientifique, UMR7104, 67404 Illkirch, France; 3 Institut National de la Santé et de la Recherche Médicale, U964, 67404 Illkirch, France; 4 Université de Strasbourg, 67081 Strasbourg, France; 5 Biochemisches Institut, Christian-Albrechts Universität Kiel, D-24118 Kiel, Germany; 6 Centre Européen d’Etude du Diabète (CEED), DIATHEC EA 7294, F-67000 Strasbourg, France; 7 Institut National de la Santé et de la Recherche Médicale U1190, EGID, CHU Lille, University of Lille, 59000 Lille, France; 8 Open Innovation Access Platform, Sanofi-Aventis R&D, 67080 Strasbourg, France; 9 European Molecular Biology Laboratory, Electron Microscopy Core Facility, 69117 Heidelberg, Germany; 10 European Molecular Biology Laboratory, Cell Biology and Biophysics Unit, 69117 Heidelberg, Germany; 11 Laboratoire de Biochimie et de Biologie Moléculaire, Nouvel Hôpital Civil, 67091 Strasbourg, France; 12 Present Address: Ludwig Institute for Cancer Research; Department of Cellular and Molecular Medicine, University of California San Diego, La Jolla, CA 92093 USA

**Keywords:** Macroautophagy, Golgi, Lysosomes, Diabetes

## Abstract

Compromised function of insulin-secreting pancreatic β cells is central to the development and progression of Type 2 Diabetes (T2D). However, the mechanisms underlying β cell failure remain incompletely understood. Here, we report that metabolic stress markedly enhances macroautophagy-independent lysosomal degradation of nascent insulin granules. In different model systems of diabetes including of human origin, stress-induced nascent granule degradation (SINGD) contributes to loss of insulin along with mammalian/mechanistic Target of Rapamycin (mTOR)-dependent suppression of macroautophagy. Expression of Protein Kinase D (PKD), a negative regulator of SINGD, is reduced in diabetic β cells. Pharmacological activation of PKD counters SINGD and delays the onset of T2D. Conversely, inhibition of PKD exacerbates SINGD, mitigates insulin secretion and accelerates diabetes. Finally, reduced levels of lysosomal tetraspanin CD63 prevent SINGD, leading to increased insulin secretion. Overall, our findings implicate aberrant SINGD in the pathogenesis of diabetes and suggest new therapeutic strategies to prevent β cell failure.

## Abstract (web summary)

Impaired beta-cell insulin secretion is a key pathological feature of type 2 diabetes. Here, the authors describe metabolic stress induced lysosomal degradation of newly formed insulin granules, independent of macroautophagy, as a potential mechanism for beta-cell dysfunction.

## Introduction

Cells employ unique evolutionary programmed strategies to cope with changes in nutrient availability. In complex organisms, these cellular responses are coordinated through hormonal cues with the aim to maintain whole-body metabolic homeostasis. The pancreatic β cell harbors an exemplary nutrient sensing machinery coupled to insulin secretion, which controls glucose homeostasis in normal conditions and is disturbed in diabetes.

Macroautophagy is a major mechanism used by the cells to remove damaged organelles and unused or aggregated proteins. In addition, it helps maintaining cellular homeostasis and is activated in response to nutrient shortage^1–3^. Macroautophagy involves formation of cargo-containing autophagosomes, which is controlled by a complex molecular machinery consisting of the autophagy-related (ATG) proteins^4^. Autophagosomes subsequently fuse with lysosomes to generate autolysosomes in which degradation occurs.

Several studies have demonstrated that macroautophagy is crucial to maintain β cell function in response to cellular stress linked to T2D. Evidence for the protective role of macroautophagy has been provided in high-fat diet-induced obesity^5,6^, in Endoplasmic Reticulum (ER) stress-induced diabetes^7,8^, in human islet amyloid polypeptide-induced diabetes^9–12^ as well as in lipo- and glucotoxicity models^13–16^. Importantly, both enhanced^5,7,15,17^ and reduced macroautophagy^13,16,18^ have been reported in metabolically challenged β cells. These seemingly controversial observations can be explained by the fact that macroautophagy levels are high and protective in compensated β cells in pre-diabetes, while they decrease at later stages, which contributes to β cell failure. In fact, depending on the nature and duration of metabolic challenges, levels of macroautophagy varied accordingly^15,19^. Finally, consistent with perturbed autophagic flux, accumulation of autophagosomes has been observed in β cells of islets isolated from T2D patients^20,21^.

Pancreatic β cells dedicate up to 50% of their protein biosynthesis to insulin production^22^, requiring a tight control of insulin granule homeostasis. While mechanisms governing insulin granule biogenesis and exocytosis in response to nutrients were widely investigated^23,24^, little is known about how nutrients impact on insulin granule turnover. Targeting of insulin granules to the lysosomes has been reported to occur in autophagosome-dependent and -independent manner^25–31^.

Recently we have described a macroautophagy-independent mechanism in pancreatic β cells as an immediate response for them to adapt to nutrient depletion^32^. We have demonstrated that nutrient-deprived β cells deliver newly synthesized secretory granules (SGs) to lysosomes in the Golgi area, where degradation of their cargo (proinsulin and insulin) provides necessary nutrients leading to mTOR-mediated suppression of macroautophagy. Starvation-induced nascent granule degradation allows β cells to cope with shortage of nutrients and to prevent unwanted insulin release. As uncontrolled lysosomal degradation of insulin granules may provoke loss of insulin and macroautophagy dysfunction, both of which occur in diabetic β cells, we wondered whether aberrant activation of this pathway might be implicated in β cell failure in T2D.

To test this hypothesis, we now focus on several models, including metabolically stressed β cell lines, human and mouse pancreatic islets as well as diabetic mice. In all of these, we find markedly increased lysosomal degradation of nascent insulin granules, which results in depletion of insulin and mTOR-dependent suppression of macroautophagy. As this pathway is not restricted to conditions of nutrient deprivation, we now refer to it as stress-induced nascent granule degradation (SINGD, pronounced ˈs**ɪ**ndi).

Furthermore, we provide evidence in vivo that pharmacological inhibition of SINGD delays onset of T2D, while activation of SINGD accelerates its progression. Finally, we find that the tetraspanin CD63 determines the destination of nascent insulin granules towards degradation versus secretion. Collectively, our findings delineate a mechanism that links impaired signaling at the Golgi to β cell failure in T2D.

## Results

### Metabolic stress enhances SINGD

**Fig. 1:**
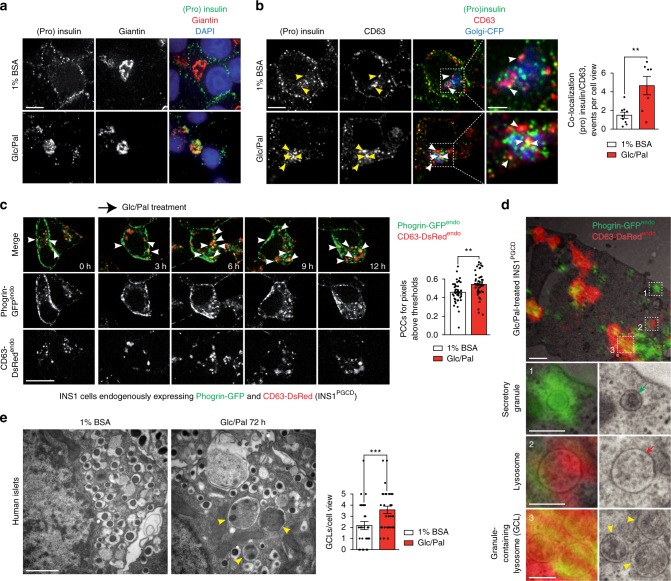
Targeting of secretory granules to lysosomes in response to glucolipotoxicity in β cells. **a** Immunofluorescence (IF) of insulin + proinsulin (“(pro)insulin”) (green) and giantin (red) in INS1 cells treated with control (1% BSA, 11 mM glucose, “1% BSA”) or glucolipotoxic (1% BSA, 33.3 mM Glucose and 0.4 mM Palmitate, “Glc/Pal”) media for 20 h. Nuclei were stained with DAPI. Scale bar, 5 μm. **b** IF of (pro)insulin (green) and CD63 (red) in INS1 cells treated with 1% BSA or Glc/Pal media for 20 h. Golgi-CFP was used to visualize the Golgi apparatus. Arrowheads point to (pro)insulin/CD63 co-localization. Scale bar, 5 μm; for insets: 2 μm. Quantification of co-localization events between (pro)insulin and CD63 per cell view. Fields from three independent experiments, *N*_1%BSA_ = 9; *N*_Glc/Pal_ = 11, *n* cells: 82 and 64, respectively; **P < 0.01, two-tailed *t*-test. **c** Imaging of INS1^PGCD^ cells endogenously expressing Phogrin-GFP (Phogrin-GFP^endo^) and CD63-DsRed (CD63-DsRed^endo^) treated with Glc/Pal for indicated time periods. Arrowheads point to Phogrin-GFP^endo^/CD63-DsRed^endo^ co-localization. Scale bar, 20 μm. Quantification of co-localization between CD63-DsRed^endo^ and Phogrin-GFP^endo^ using Pearson’s correlation. *N*_1%BSA_ = 42; *N*_Glc/Pal_ = 50 from three independent experiments; **P < 0.01, two-tailed *t*-test. **d** Correlative light and electron microscopy analysis of INS1^PGCD^ cells treated with Glc/Pal for 20 h. Yellow arrowheads point to granule-containing lysosomes (GCLs). Green and red arrows point to a secretory granule and a lysosome respectively. Scale bar, 1 μm; for insets: 0.3 μm. **e** Electron microscopy analysis of β cells in human islets treated with 1%BSA or Glc/Pal media for 72 h. Yellow arrowheads point to GCLs. Scale bar, 1 μm. Quantification of GCLs per cell view. *N*_1%BSA_ = 29; *N*_Glc/Pal_ = 31 from three independent experiments; ***P < 0.001, two-tailed *t*-test. In **b**, **c**, **e** data are shown as mean ± SEM, source data are provided as a Source Data file

We first investigated SINGD in β cells upon chronic exposure to elevated levels of Glucose (Glc) and Palmitate (Pal), i.e., glucolipotoxic conditions^33^. We treated rat insulinoma-derived β cells (INS1 cells) with 33.3 mM Glc and 0.4 mM Pal (Glc/Pal) or 11 mM Glc (control growing culture) for 20 h. We observed that Glc/Pal led to a drastic decrease in insulin-positive SGs at the plasma membrane in comparison to control conditions. Importantly, Glc/Pal-treated cells also accumulated SGs in the Giantin-positive Golgi area (Fig. 1a, Supplementary Fig. 1a) prompting us to further characterize the latter. Immunofluorescence of proinsulin/insulin ((pro)insulin) and CD63 as markers of SGs and late endosomes/lysosomes respectively, revealed abundant large CD63 puncta, co-localizing with (pro)insulin in the Golgi area in Glc/Pal-treated INS1 in comparison to control cells (Fig. 1b). Accordingly, increased co-localization of (pro)insulin with another lysosomal marker Lysosomal-Associated Membrane Protein 2 (LAMP2) was found in the Golgi area upon Glc/Pal as compared to control treatment (Supplementary Fig. 1b). Collectively, these findings hint at potential induction of SINGD upon glucolipotoxic conditions.

To address the dynamics of SINGD in metabolically challenged cells, we generated INS1 cells endogenously expressing the SG marker Phogrin tagged with Green Fluorescent Protein (GFP) and the lysosomal marker CD63 tagged with *Discosoma sp*. Red fluorescent protein (DsRed) (hereafter referred to as INS1^PGCD^ cells). Using CRISPR-Cas9 gene editing^34^, we added sequences coding corresponding fluorescent proteins to the 3′ end of the last exon of *PTPRN2* (or Phogrin) and *CD63*, respectively (Supplementary Fig. 2a–j). INS1^PGCD^ cells contain a homozygous insertion of GFP in the Phogrin locus, and a heterozygous insertion of DsRed in the CD63 locus, confirmed by Sanger sequencing and PCR analysis (Supplementary Fig. 2b, d, g, h). The expression of Phogrin-GFP^endo^ was corroborated by immunobloting (Supplementary Fig. 2c) and cellular localization of Phogrin-GFP^endo^ and CD63-DsRed^endo^ was confirmed by immunofluorescence and correlative light and electron microscopy (CLEM) (Supplementary Fig. 2e, i, j). In line with SINGD triggered by starvation^32^, INS1^PGCD^ cells demonstrated increased Phogrin/CD63 co-localization upon nutrient deprivation as compared to control conditions (Supplementary Fig. 3a). Accordingly, inhibition of Protein Kinase D (PKD), a major negative regulator of SINGD^32^, using the selective PKD inhibitor CID755673^35^ also resulted in increased Phogrin/CD63 co-localization (Supplementary Fig. 3b). Time-lapse microscopy of INS1^PGCD^ cells treated with Glc/Pal revealed a marked increase in Phogrin/CD63 co-localization starting already after 6 h of treatment (Fig. 1c). Interestingly, INS1^PGCD^ cells treated separately with Glc or Pal also demonstrated increased Phogrin/CD63 co-localization indicating that various inducers of metabolic stress are capable to trigger SINGD (Supplementary Fig. 3c).

We next tested whether SINGD induced by Glc/Pal occurred via macroautophagy. To this end, we followed SG markers, (pro)insulin and Phogrin, in INS1^LC3B-GFPendo^ cells endogenously expressing LC3B tagged with GFP^32^, a marker for autophagic intermediate compartments – autophagosomes^36^. While we observed occasional autophagosomes containing SGs in control-treated cells, the amount of SG-positive autophagosomes was almost abolished upon Glc/Pal treatment, suggesting that SINGD does not rely on macroautophagy (Supplementary Fig. 4a, b).

We next monitored SINGD upon downregulation of the major canonical macroautophagy genes *atg5* and *beclin1 (becn1)* that play a crucial role in early steps of the macroautophagy pathway by controlling the biogenesis of autophagosomes^4^. The lysosomal v-ATPase inhibitor Bafilomycin A1 (BafA1) counters lysosome activity and prevents the fusion of autophagosomes with lysosomes^37,38^, and thus is routinely used to measure autophagic flux. As expected, silencing of *atg5* and *beclin1* led to a drastic decrease in the number of LC3B-GFP-positive puncta in BafA1-treated Glc/Pal-treated INS1^LC3B-GFPendo^ cells, indicating that early steps of macroautophagy were inhibited prior formation of autophagosomes (Supplementary Fig. 4c, d). However, in line with macroautophagy-independent SINGD^32^, silencing of *atg5* and *beclin1* did not lead to any decrease in Phogrin/CD63 co-localization upon Glc/Pal (Supplementary Fig. 4e–g).

Importantly, BafA1 markedly increased the amount of LC3B-positive/CD63-negative puncta in Glc/Pal-treated INS1^PGCD^ cells, in line with accumulation of autophagosomes upon inhibition of fusion of autophagosomes with lysosomes (Supplementary Fig. 4h). If Glc/Pal-induced delivery of SGs to lysosomes occurred via autophagosomes, BafA1 treatment would lead to accumulation of SG-containing autophagosomes in the cytoplasm, preventing delivery to CD63-positive lysosomes. However, we observed the opposite: the LC3B-positive/CD63-negative autophagosomes did not contain SGs; and the size and amount of co-localized Phogrin/CD63 signals was further increased upon BafA1, overall corroborating macroautophagy-independent SINGD (Supplementary Fig. 4i, j).

We next used correlative light and electron microscopy (CLEM) to follow SINGD at the ultrastructural level. First, CLEM of Glc/Pal-treated INS1^PGCD^ cells confirmed large CD63- and Phogrin-positive granule-containing lysosomes (GCLs) (Fig. 1d and Supplementary Fig. 5a). Second, live-cell imaging followed by CLEM (live-CLEM) identified that GCLs were formed via direct fusion between SGs and lysosomes (Supplementary Fig. 5b and Supplementary Movie 1).

Finally, β cells of primary human islets, treated with Glc/Pal for 72 h contained abundant GCLs in the Golgi area, as revealed by quantitative Electron Microscopy (EM) analysis (Fig. 1f). Altogether, our data indicate that prolonged exposure of β cells to Glc/Pal diverts SGs from the secretory route to lysosomes in a macroautophagy-independent manner.

### mTOR suppresses macroautophagy upon metabolic stress

**Fig. 2:**
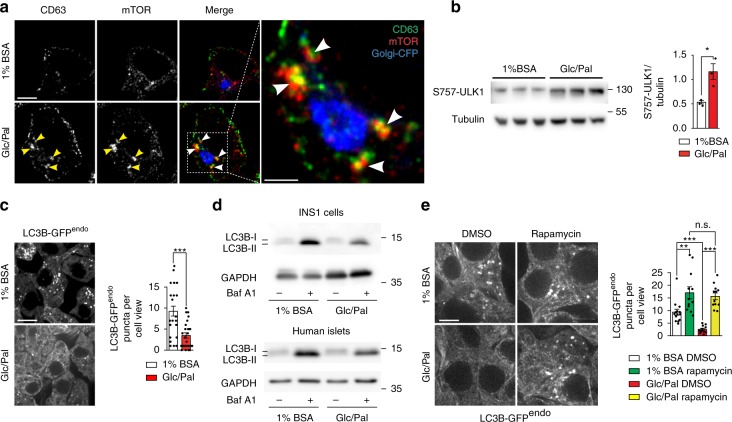
Glucolipotoxicity leads to mTOR-dependent inhibition of macroautophagy in β cells. **a** Immunofluorescence of CD63 (green) and mTOR (red) in INS1 cells treated with control (1% BSA, 11 mM glucose, “1% BSA”) or glucolipotoxic (1% BSA, 33.3 mM Glucose and 0.4 mM Palmitate, “Glc/Pal”) media for 20 h. Golgi-CFP was used to visualize the Golgi apparatus. Arrowheads point to large CD63/mTOR puncta. Scale bar, 5 μm; for inset: 2 μm. 3D reconstruction is shown in Supplementary Movie 2. **b** Immunoblot of S757-ULK1 using lysates of INS1 cells treated with 1% BSA or Glc/Pal media for 48 h. Tubulin was used as a loading control. Quantification of S757-ULK1/Tubulin ratio. *N* = 3 per group; *P < 0.05, two-tailed *t*-test. **c** LC3B-GFP^endo^ puncta in INS1 cells endogenously expressing LC3B-GFP (INS1^LC3B-GFPendo^) treated with 1% BSA or Glc/Pal media for 40 h. Scale bar, 10 μm. Quantification of the puncta per cell view, two independent experiments, *N*_1%BSA_ = 24; N_Glc/Pal_ = 26; ***P < 0.001, two-tailed *t*-test. **d** Immunoblot of LC3B using lysates of INS1 cells (top) or human islets (bottom) treated with 1% BSA or Glc/Pal for 40 h in the presence or absence of bafilomycin A1 (BafA1), 10 nM added for the last 1 h (INS1) or 2 h (islets) of incubation. GAPDH was used as a loading control. **e** LC3B-GFP^endo^ puncta in INS1^LC3B-GFPendo^ cells treated for 40 h as indicated, DMSO or rapamycin (100 nM) were added for the last 2 h of incubation. Scale bar, 5 μm. Quantification of puncta per cell view. Fields from three independent experiments, *N*_1%BSA DMSO_ = 14; *N*_1%BSA Rapamycin_ = 12; *N*_Glc/Pal DMSO_ = 12; *N*_Glc/Pal Rapamycin_ = 12; *n* cells: 50, 32, 32, 28 respectively; **P < 0.01, ***P < 0.001, n.s.: not significant, two-tailed *t*-test. In **b**, **c**, **e** data are shown as mean ± SEM, source data are provided as a Source Data file

Activation of mammalian/mechanistic Target of Rapamycin (mTOR) Complex 1 occurs at the lysosomal membrane in response to addition of amino acids^39–41^. We have recently shown that SINGD triggered by starvation was associated with increased recruitment of mTOR to GCLs, mTOR activation and suppression of macroautophagy via mTOR-mediated inhibitory phosphorylation of Unc-51–like kinase 1 (ULK1)^32^. We thus next asked whether nutrient stress imposed by Glc/Pal treatment evoked similar effects. In fact, prolonged Glc/Pal treatment was shown to induce macroautophagy dysfunction in a mTOR-dependent manner^13,16^. We observed that Glc/Pal recruited mTOR to CD63-positive lysosomes in INS1 cells (Fig. 2a, Supplementary Movie 2) and increased phospho-ULK1 (Fig. 2b). Moreover, INS1^LC3B-GFPendo^ cells treated with Glc/Pal for 20 h contained less LC3B-GFP puncta as compared to control-treated cells (Fig. 2c). Accordingly, immunoblotting revealed a decrease in lipidated autophagosomal LC3B-II in Glc/Pal-treated INS1 cells and primary human islets as compared to control conditions. This difference was evident in the presence and absence of BafA1, indicating reduced autophagy flux (Fig. 2d, quantified in Supplementary Fig. 6). Consistent with mTOR-mediated suppression of macroautophagy in Glc/Pal-treated cells, the mTOR inhibitor rapamycin increased LC3B-GFP puncta in Glc/Pal-treated INS1^LC3B-GFPendo^ cells (Fig. 2e). Altogether, these data indicate that lysosomal degradation of SGs is likely to contribute to mTOR-dependent suppression of macroautophagy in metabolically stressed β cells.

### Increased GCLs and decreased macroautophagy in diabetic mice

We have identified enhanced SINGD in β cell lines and primary islets treated under gluco- and lipotoxic conditions. To extend and confirm these findings in vivo we subjected mice to a metabolic stress by feeding animals a high-fat diet (HFD) for 13 weeks (Supplementary Fig. 7a, b), and analyzed the frequency of fusion events between lysosomes and SGs in β cells. In line with above findings, EM and IF analysis of β cells of HFD-fed mice revealed higher numbers of GCLs, as well as co-localized (pro)insulin/CD63 indicating increased SINGD (Supplementary Fig. 7c, d).

**Fig. 3:**
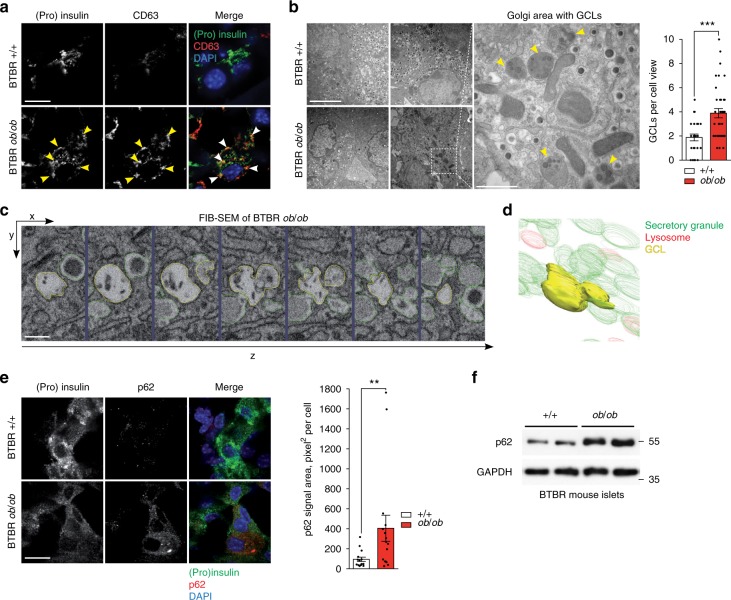
Increased granule-containing lysosomes (GCLs) and decreased macroautophagy in diabetic mice. **a** Immunofluorescence (IF) of insulin + proinsulin ((pro)insulin) (green) and CD63 (red) in β cells of 8-week-old BTBR +/+ and BTBR *ob*/*ob* mice. Arrowheads point to (pro)insulin/CD63 co-localization. Scale bar, 20 μm. **b** Electron microscopy of β cells in pancreatic islets isolated from 6-week-old BTBR +/+ and BTBR *ob*/*ob* mice. Yellow arrowheads point to granule-containing lysosomes (GCLs). Scale bar, 5 μm; for inset: 1 μm. Quantification of GCLs per cell view. *N*_+/+_ = 25 cells and *N*_*ob*/*ob*_ = 36 cells from three mice per each group. ***P < 0.001, Mann–Whitney *U*-test. **c** Focused Ion Beam Scanning Electron Microscopy (FIB-SEM) of GCLs of 6-week-old BTBR *ob*/*ob* mice. Sequential Z-slices showing GCL (yellow) and neighboring insulin granules (green). Scale bar, 0.2 μm. **d** 3D reconstruction of the GCL revealed by FIB-SEM in **c**. Detailed 3D reconstruction is shown in Supplementary Movie 3. **e** IF of (pro)insulin (green) and p62 (red) in β cells of 8-week-old BTBR +/+ and BTBR *ob*/*ob* mice. Scale bar, 20 μm. Quantification of p62 signal area: area of p62 signal (pixel²) over a fixed signal intensity threshold. *N*_+/+_ = 15; *N*_*ob*/*ob*_ = 16, from three mice per each group. **P < 0.01, Mann-Whitney U-test. **f** Immunoblot of p62 using lysates of islets isolated from 8-week-old BTBR +/+ and BTBR *ob*/*ob* mice. GAPDH was used as a loading control. In **b** and **e** data are shown as mean ± SEM, source data are provided as a Source Data file. (**a**, **e**) Nuclei were stained with DAPI

We then decided to study SINGD in a model of T2D, leptin-deficient *ob*/*ob* mice in a BTBR genetic background (BTBR^*ob*/*ob*^)^42^. The BTBR^*ob*/*ob*^ mice develop severe hyperglycemia and diabetes between 6 and 8 weeks of age^42^ (Supplementary Fig. 8a, b). β- cells in islets of 8-week-old BTBR^*ob*/*ob*^ mice contained less (pro)insulin-positive puncta in comparison to non-diabetic control mice (BTBR^+/+^ mice). Strikingly, these residual puncta largely co-localized with CD63 (Fig. 3a). Furthermore, quantitative EM confirmed reduced SG abundance and revealed a marked increase in GCLs in the Golgi area of β cells in islets of 6-week-old BTBR^*ob*/*ob*^ mice as compared to control mice (Fig. 3b). Finally, 3D EM reconstruction using Focused Ion Beam Scanning EM corroborated abundant GCLs in Golgi area of diabetic β cells (Fig. 3c, d and Supplementary Movie 3).

As SINGD counters macroautophagy, we next analyzed macroautophagy levels in control and diabetic β cells. p62 binds polyubiquitinated protein aggregates, mediating their clearance by autophagy^43^. Cytosolic accumulation of enlarged p62-positive inclusions has been described in autophagy-deficient β cells^44^. Consistently, β cells of 8-week-old BTBR^*ob*/*ob*^ mice contained large p62 puncta, suggesting compromised autophagy (Fig. 3e). Increased p62 levels in islets of BTBR^*ob*/*ob*^ mice were further corroborated by immunoblotting (Fig. 3f). Importantly, p62 protein accumulation in islets of BTBR^*ob/ob*^ mice was not due to increased p62 mRNA levels (Supplementary Fig. 9).

### Enhanced SINGD leads to β cell dysfunction in diabetes

**Fig. 4:**
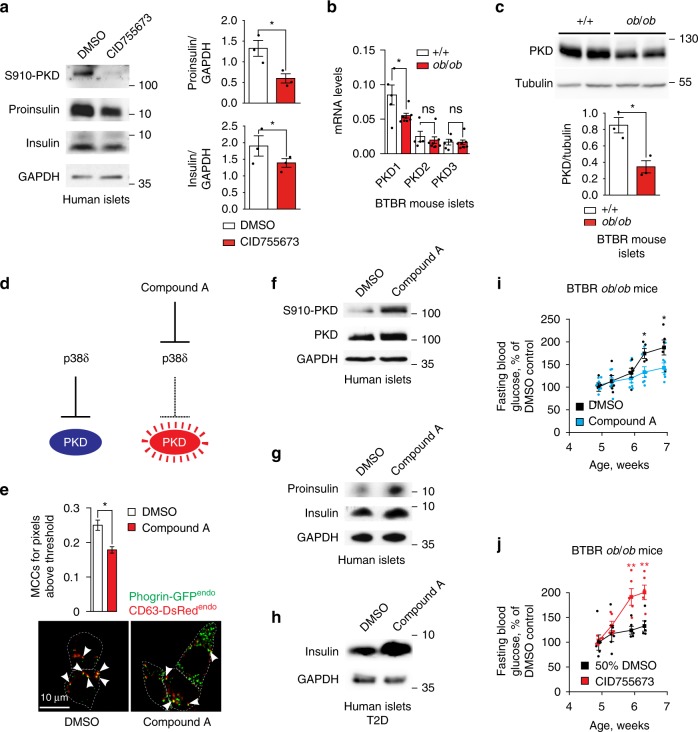
SINGD contributes to β cell dysfunction in diabetes. **a** Immunoblot of indicated proteins using lysates of isolated human islets treated with DMSO or PKD inhibitor CID755673 (30 μM) for 24 h. GAPDH was used as a loading control. Quantification of Proinsulin/GAPDH and Insulin/GAPDH ratios, *P < 0.05, *N* = 3, one-tailed, paired *t*-test. **b** Quantitative RT-PCR using mRNA isolated from pancreatic islets of 8-week-old BTBR +/+ and BTBR *ob*/*ob* mice using primer pairs amplifying cDNA of PKD1, PKD2 and PKD3. The expression values were normalized to housekeeping genes HPRT and GAPDH. *N*_+/+_ = 8; *N*_*ob*/*ob*_ = 5; *P < 0.05, two-tailed *t*-test. **c** Immunoblot of PKD using lysates of islets isolated from 8-week-old BTBR +/+ and BTBR *ob*/*ob* mice. Tubulin was used as a loading control. Quantification of PKD/Tubulin ratio, *P < 0.05, *N* = 3 per group, two-tailed *t*-test. **d** Compound A activates PKD through inhibition of p38δ. **e** Imaging of INS1^PGCD^ cells endogenously expressing Phogrin-GFP(Phogrin-GFP^endo^) and CD63-DsRed (CD63-DsRed^endo^) treated with Glc/Pal for 24 h in the presence of DMSO or Compound A, 100 nM. Arrowheads point to Phogrin-GFP^endo^/CD63-DsRed^endo^ co-localization. Scale bar, 10 μm. Quantification of co-localization between CD63-DsRed^endo^ and Phogrin-GFP^endo^ using Manders coefficients. *N*_Glc/Pal,DMSO_ = 58; *N*_*G*lc/Pal,Compound A_ = 59 from three independent experiments. *P < 0.01, two-tailed *t*-test. **f**, **g** Immunoblots of indicated proteins using lysates of isolated human islets treated with DMSO or Compound A for 48 h. GAPDH was used as a loading control. **h** Immunoblot of indicated proteins using lysates of human islets isolated from a T2D donor. Islets were treated with DMSO or Compound A for 48 h. GAPDH was used as a loading control. **i** Time-course of fasting blood glucose levels of BTBR *ob*/*ob* mice of indicated ages implanted with osmotic pumps containing control solvent (DMSO) or Compound A. *N*_DMSO_ = 5; *N*_Compound A_ = 7; *P < 0.05, two-tailed *t*-test. **j** Time-course of fasting blood glucose levels of BTBR *ob*/*ob* mice of indicated ages implanted with osmotic pumps containing control solvent (50% DMSO) or CID755673. *N*_50% DMSO_ = 5; *N*_CID755673_ = 5; **P < 0.01, two-tailed *t*-test. In **a**, **b**, **c**, **e**, **i**, and **j** data are shown as mean ± SEM, source data are provided as a Source Data file

Our findings provide strong evidence for increased SINGD in diabetic β cells. However, it is unclear whether SINGD observed upon Glc/Pal treatment as well as in diabetic or metabolically stressed mice contributes to β cell failure *per se* or represents a secondary event - for example, a removal of excessive SGs due to perturbed insulin secretion. To distinguish between these possibilities, it is necessary to follow SGs trafficking and release in conditions where SINGD is prevented. We have previously found that SINGD is controlled by PKD1: while inhibition or decreased expression of PKD1 induces SINGD, high PKD1 activity counters SINGD^32^. Indeed, induction of SINGD by inhibition of PKD resulted in a decrease of insulin and proinsulin in human pancreatic islets (Fig. 4a). Interestingly, consulting a published study comparing genome-wide mRNA expression in non-diabetic BTBR^+/+^ and diabetic BTBR^*ob*/*ob*^ mice^45^, revealed a decrease in PKD1 mRNA abundance in diabetic islets (Supplementary Fig. 10). Similarly, in line with decreased PKD1 triggering SINGD in diabetic β cells, we found a marked reduction in mRNA and protein levels of PKD1 in islets of diabetic 8-week-old BTBR^*ob*/*ob*^ mice as compared to age-matched control mice (Fig. 4b, c).

We thus decided to test whether PKD activation prevented SINGD in diabetes. Mitogen-Activated Protein Kinase (MAPK) p38δ negatively regulates PKD1, and loss or reduction of p38δ results in increased activity of PKD1, which counters SINGD^32,46^. We generated a Compound A, which selectively inhibited p38δ and activated PKD (Fig. 4d, Supplementary Fig. 11a–c). Compound A decreased GCLs and increased SGs in Glc/Pal-treated INS1^PGCD^ cells (Fig. 4e). Furthermore, phospho-PKD, proinsulin and insulin levels were increased in Compound A-treated human pancreatic islets isolated from non-diabetic donors (Fig. 4f, g, quantified in Supplementary Fig. 11d). In addition, Compound A increased insulin levels in islets isolated from T2D patients (Fig. 4h, quantified in Supplementary Fig. 11d).

Importantly, while p38δ is abundantly expressed in pancreas, no expression of p38δ has been detected in insulin-sensitive organs such as adipose tissue and liver^46^, prompting us to address the effects of Compound A on SINGD in vivo. We thus infused Compound A or a control solution into 4-week-old BTBR^*ob*/*ob*^ mice for 2 weeks using osmotic pumps and followed blood glucose levels over time. As expected, fasting blood glucose levels progressively increased in control-treated BTBR^*ob*/*ob*^ mice, indicating the onset of diabetes. However, glucose levels in Compound A-treated BTBR^*ob*/*ob*^ mice were lower than in control-treated animals indicating protection against diabetes (Fig. 4i). In strong contrast, systemic inhibition of PKD using the compound CID755673 accelerated onset of diabetes in BTBR^*ob*/*ob*^ mice, as evidenced by increased fasting blood glucose (Fig. 4j) and decreased insulin secretion in response to glucose injections (Supplementary Fig. 12a). Strikingly, upon islet isolation, it was evident that islets from CID755673-treated mice were pale to transparent as compared to yellow to golden islets from control-treated mice (Supplementary Fig. 12b), a phenotype hinting at the loss of insulin^47^. Accordingly, insulin levels were markedly decreased in islets of CID755673-treated mice (Supplementary Fig. 12c). In contrast, the numbers of GCLs were increased in β cells of islets isolated from CID755673-treated BTBR^*ob*/*ob*^ mice as compared to control solution-treated BTBR^*ob*/*ob*^ mice (Supplementary Fig. 12d).

Collectively, these data suggest that reducing PKD activity in vivo is sufficient to trigger SINGD in β cells, while PKD activity prevents SINGD and protects against β cell dysfunction. Accordingly, the observed reduced PKD expression in diabetic islets is likely to contribute to enhanced SINGD in β cells in diabetes. Even though a direct implication of enhanced SINGD to β cell failure in vivo is to be substantiated, its link to reduced insulin content, compromised macroautophagy and insulin secretion is suggestive for such a scenario.

### Stress-induced nascent granule degradation requires CD63

**Fig. 5:**
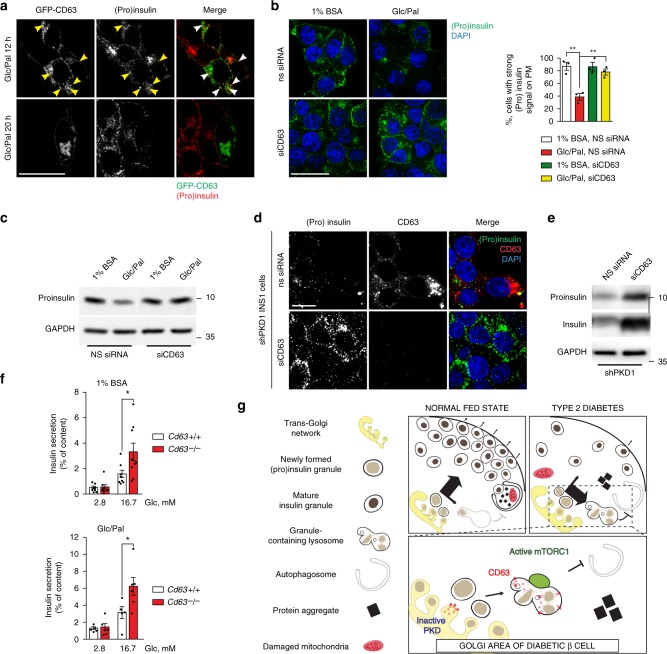
Stress-induced nascent granule degradation requires CD63. **a** Immunofluorescence (IF) of insulin + proinsulin (”(pro)insulin”) (red) in INS1 cells overexpressing GFP-CD63 (green) and treated with control (1% BSA, 11 mM glucose, “1% BSA”) or glucolipotoxic (1% BSA, 33.3 mM Glucose and 0.4 mM Palmitate, “Glc/Pal”) media for the indicated time. Arrowheads point to GFP-CD63/(pro)insulin co-localization. Scale bar, 20 μm. **b** IF of (pro)insulin (green) in INS1 cells transfected with non-silencing (ns) siRNA or siRNA against CD63 (siCD63) and treated with 1% BSA or Glc/Pal for 20 h. Scale bar, 20 μm. Quantification of (pro)insulin signals at the plasma membrane of INS1 cells treated as indicated. Three independent experiments (*N* = 3), three fields per experiment per condition, *n* cells: 63, 62, 86, 71 respectively; **P < 0.01, two-tailed *t*-test. **c** Immunoblot of proinsulin using lysates of INS1 cells treated as indicated. GAPDH was used as a loading control. **d** IF of (pro)insulin (green) and CD63 (red) in INS1 cells stably expressing shRNA against *Prkd1* (shPKD1), transfected with indicated siRNAs for 48 h. Scale bar, 10 μm. **b**, **d** Nuclei were stained with DAPI. **e** Immunoblot of proinsulin and insulin using lysates from INS1 cells stably expressing shPKD1, transfected with indicated siRNAs. GAPDH was used as a loading control. **f** Glucose stimulated insulin secretion (2.8 mM Glc and 16.7 mM Glc) of islets isolated from *Cd63* +/+ or *Cd63* −/− mice pre-treated with control (1% BSA) or glucolipotoxic (Glc/Pal) media for 48 h. Batches of islets were independently isolated from *Cd63* +/+ and *Cd63* −/− mice; *N*_1% BSA_ = 8 (*Cd63* +/+ ), 9 (*Cd63*−/−), *N*_Glc/Pal_ = 5 (*Cd63* +/+ ), 6 (*Cd63* −/−), *P < 0.05, two-tailed *t*-test. In **b** and **f** data are shown as mean ± SEM, source data are provided as a Source Data file. **g** Stress-induced nascent granule degradation (SINGD) in Type 2 Diabetes (T2D). In normal fed state, PKD1 promotes secretory granule biogenesis and sorting towards secretion, SINGD is low. However, in T2D, a decrease in PKD1 leads to the CD63-dependent targeting of the newly formed granules to the CD63-positive lysosomes instead of secretion. As a result, mTORC1 is recruited to the membrane of the granule-containing lysosomes, and its chronic activation leads to inhibition of the housekeeping process of macroautophagy. Thus, aberrant activation of SINGD contributes to β cell failure in T2D

Our above findings reveal that reduced PKD activity triggers SINGD, while enhanced PKD activity prevents SINGD. Mechanistically, SINGD is manifested as delivery of SGs to CD63-positive lysosomes in Glc/Pal-treated β cells and in β cells of BTBR^*ob*/*ob*^ mice. Interestingly, we noticed that transient overexpression of CD63 resulted in a marked depletion of SGs upon Glc/Pal treatment, suggesting that CD63 might mediate SINGD (Fig. 5a). Of note, while the deletion of the lysosomal proteins LIMP2 and LAMP2 was associated with severe lysosomal dysfunction^48,49^, mice lacking CD63 demonstrated normal overall lysosome function^50^. Therefore, we decided to investigate secretion and degradation of SGs in the absence of CD63. To address the role of CD63 in SINGD, we first transfected INS1 cells with siRNA against *Cd63* or non-silencing (NS) siRNA and incubated cells in the media with or without Glc/Pal for 20 h. In control cells, transfected with NS siRNA, Glc/Pal treatment led to a decrease in (pro)insulin signals in the plasma membrane area. In contrast, SG localization remained largely unchanged in Glc/Pal-treated CD63-knockdown cells (Fig. 5b), indicating that SINGD was mediated by CD63. Furthermore, immunoblotting revealed a drop in proinsulin levels upon Glc/Pal treatment in control-transfected cells, but not in CD63-knockdown cells (Fig. 5c). To directly test whether SINGD induced by reduction of PKD requires CD63, we examined SINGD in INS1 cells stably expressing shRNA against *Prkd1* (shPKD1) in the presence and absence of CD63. As expected, INS1 cells lacking PKD1 contained few SGs and displayed large CD63 puncta co-localizing with residual SGs (Fig. 5d). However, siRNA-mediated depletion of CD63 from PKD1-knockdown cells prevented loss of SGs, indicating that CD63 mediates PKD1-controlled SINGD (Fig. 5d, e). Our above findings suggest that depletion of CD63 allows SGs to escape degradation and to reach the plasma membrane, possibly for secretion. Therefore, lowering CD63 levels is expected to increase insulin secretion. To address the latter point, we next compared insulin secretion from islets derived from *Cd63* knockout (*Cd63*^−/−^ mice)^50^ and wild-type *Cd63*^+/+^ mice. Consistent with reduced SINGD, quantitative EM analysis revealed a decrease in the number of GCLs in the Golgi area, and an increase in the number of SGs associated with the plasma membrane in β cells of islets isolated from *Cd63*^−/−^ mice (Supplementary Fig. 13a, b). To test the effects of *Cd63* deletion on insulin secretion upon a metabolic challenge, we also treated *Cd63*^+/+^ and *Cd63*^−/−^ islets with Glc/Pal for 48 h. While this treatment did not abrogate GSIS in control islets, it led to a decrease in total insulin content, most likely due to increased secretion of insulin during incubation with Glc/Pal (Supplementary Fig. 13c). Strikingly, *Cd63*^−/−^ islets demonstrated a two-fold increase in GSIS under both control and Glc/Pal treatment conditions (Fig. 5f). Total insulin levels were similar in metabolically challenged *Cd63*^+/+^ and *Cd63*^−/−^ islets (Supplementary Fig. 13c), suggesting that SGs escaped from SINGD in *Cd63*^−/−^ β cells and instead were secreted.

Taken together, our data demonstrate that β cells developed complex machinery at the Golgi that controls targeting of insulin granules towards secretion or lysosomal degradation. Nutrient stress-imposed PKD deregulation triggers SINGD at least partially in a CD63-dependent manner. This mechanism may contribute to insulin loss, reduced secretory capacity and to decreased macroautophagy, hallmarks of β cell dysfunction in T2D (Fig. 5g).

## Discussion

Our findings in β cell lines endogenously expressing markers of SGs and lysosomes, in human and mouse islets, as well as in mouse models identified the SINGD pathway as an essential mechanism that may contribute to both loss of insulin granules and impaired macroautophagy, key features of β cell dysfunction in T2D^5,11,12,44,51–54^. To date, several mechanisms contributing to the depletion of insulin in β cells in T2D patients and in response to glucolipotoxicity have been reported, including impaired insulin gene transcription^55,56^ and mRNA stability^57–59^; Endoplasmic reticulum-associated degradation (ERAD) and other proteasomal degradation routes^60^; as well as proinsulin processing and sorting^61,62^ and basal hypersecretion^63^. SINGD can now be considered as a new mechanism with potential to contribute to insulin depletion, but whether SINGD constitutes a major pathway leading to loss of insulin in T2D will require its quantification versus the other possibilities in future studies.

It is also important to mention that our results do not exclude the possibility that a small portion of byproducts of degradation might be released from cells. For example, aberrant lysosomal secretion might be an additional potential mechanism of insulin loss induced by SINGD. Interestingly, proinsulin, the major cargo in nascent granules, is released in higher amounts in diabetic patients^64,65^. Furthermore, recent studies identified that partially degraded granule proteins including products of insulin degradation might be released from diabetic β cells^66,67^.

Several potential mechanisms leading to impaired β cell macroautophagy in T2D have been proposed, including defects in autophagosome/lysosome fusion^13,16,18,19^ and more broadly, primary defects in lysosomal function^20^. Our data hint at impaired β cell macroautophagy in T2D as a consequence of mTOR-dependent reduction in autophagosome formation in response to increased SINGD. Importantly, recent studies have also established the role of mTOR in more distal steps of macroautophagy^68,69^ suggesting that SINGD-mTOR-induced inhibition of macroautophagy may not be limited to reducing autophagosome formation.

Genetically induced mTORC1 hyperactivity has been reported to be deleterious and to promote onset of diabetes in vivo due to compromised macroautophagy^52^. Importantly, chronic mTORC1 inactivation also led to rapid onset or aggravation of diabetes^70,71^. Mechanistically, mTORC1 inactivation mainly impaired β cell compensation in response to metabolic stress. Together with our findings, these data argue for a model in which nutrient-dependent switching between high and low mTORC1 activity promoting β cell growth/proliferation or macroautophagy, respectively, is crucial to maintain β cell function^72^. This model is also supported by a recent report demonstrating that intermittent fasting promoted resistance against ageing and diabetes^73^.

Our previous work unveiled that β cells utilize SINGD in response to nutrient deprivation, thereby suppressing macroautophagy^32^. An aberrant activation of SINGD in diabetic conditions somehow parallels one of the hallmarks of T2D, which is constitutive induction of fasting-related processes, i.e., gluconeogenesis and lipolysis, in a situation of nutrient overload. Although the latter phenomena are related to insulin resistance^74^ as well as hyperglucagonaemia^75^, it remains unclear, how nutrient deprivation and nutrient excess both can trigger SINGD in β cells. It will be important to understand mechanistically how these seemingly opposing nutrient stresses both converge into reduced PKD activity at the TGN triggering SINGD.

Studies from our group and others have previously implicated perturbed Protein Kinase D activity in the pathogenesis of T2D^46,76–79^. Furthermore, PRKD1 (human PKD) was identified in several genome-wide associated studies as a Body Mass Index- associated locus^80–82^ as well as T2D-associated locus^83^. Our findings establish decreased PKD as a trigger for SINGD in diabetic β cells of mice and humans, thus suggesting a mechanistic explanation for compromised PKD activity contributing to T2D.

Our study also provides evidence for tetraspanin CD63 to be an essential component for targeting insulin granules to the lysosomes (Fig. 5g). Although high CD63 levels induce SINGD, depletion of CD63 in β cell lines and in islets prevents SINGD, restoring the pool of SGs. CD63 has been previously implicated in the sorting and targeting of various substrates to the endolysosomal system^84–86^. How does PKD activity limit CD63-mediated SINGD? According to one potential scenario, granules routed to lysosomes may lack PKD-dependent signals generated at the Golgi that prevent CD63-dependent fusion with lysosomes. In other words, CD63 might be a necessary component on lysosomes independent of PKD activity but whether or not fusion occurs might be dictated by PKD-dependent signals on the insulin granule. Alternatively, PKD may also more directly inhibit CD63 function on lysosomes or other lysosomal proteins that are required for fusion to occur. Future efforts will be necessary to test the above models. Hence, unraveling of detailed molecular mechanisms of SINGD will be required to address a more direct physiological impact of the SINGD pathway on β cell function and glucose homeostasis in vivo.

What is the place of SINGD in the context of known and putative degradation mechanisms? Our findings demonstrate that SINGD occurs via direct fusion of nascent SGs with lysosomes. Seminal electron microscopy studies performed in various secretory cells, including cells of the anterior pituitary gland^87^, pancreatic α cells^88^, and β cells^89^ had presented strong evidence for selective fusion between secretory granules and lysosomes, which in some cases correlated with conditions of perturbed secretion. This process was designated as crinophagy to distinguish it from canonical autophagy (macroautophagy)^90^. Csizmadia et al. have recently presented an extensive analysis of molecular mechanisms of crinophagy in the salivary gland of Drosophila, further highlighting the evolutionary importance of this pathway independent of macroautophagy^91^. The relevance of these findings in the context of p38δ/PKD- and CD63-dependent SINGD remains to be established.

Recently, a study by Riahi et al. performed in β cells overexpressing markers of SGs, macroautophagy as well as lysosomes claimed that macroautophagy was a major pathway through which insulin granules were degraded^30^. According to our data, this is not the case for SINGD. Yet, none of our findings contradicts the possibility that insulin SGs may eventually be delivered to lysosomes via autophagosomes along with other cytosolic material. Indeed, double-membrane autophagosomes containing SGs have been reported to occur in β cells^28^. Importantly however, a large body of evidence in our and other studies demonstrates that targeting of secretory granules to lysosomes can occur independent of canonical macroautophagy^25,26,28,31,32,87,91^. Furthermore, if macroautophagy was mainly responsible for SGs degradation, macroautophagy-deficient β cells would be expected to accumulate SGs, but not to lose them as observed by Riahi et al. and in other studies^5,30,44^. In addition, consistent with our own findings, Riahi et al. observed that treatment of β cells with BafA1 resulted in marked increase in co-localization of insulin granules with lysosomes. Among other effects, BafA1 blocks fusion of autophagosomes with lysosomes^37,38^ (Supplementary Fig. 4h) which prevents autophagosomal delivery of insulin granules to lysosomes. Finally, our data strongly indicate that SINGD induced by glucolipotoxicity does not require the macroautophagy machinery, as silencing of major macroautophagy genes *atg5* and *beclin1* has virtually no effect on SINGD.

Interestingly, a recent study has reported that secretory granules might be delivered to lysosomes via autophagosome-like structures originating at the Golgi in β cells devoid of factors required for autophagosome formation (ATG5 and ATG7)^31^. Strikingly, both Golgi membrane-associated degradation (GOMED) and SINGD occur in lysosomes adjacent to the Golgi area independent of the classical macroautophagy machinery. However, three important aspects distinguish GOMED from direct fusion of insulin granules with lysosomes as a mechanism underlying SINGD: (1) GOMED is a feature in cells lacking the canonical macroautophagy machinery. (2) Direct fusion of insulin granules with lysosomes does not rely on autophagosome-like intermediates to target SGs to lysosomes. (3) GOMED is not restricted to degradation of insulin granules but is also a way to target for example mitochondria to lysosomal degradation in the absence of canonical macroautophagy.

Collectively, the delivery of insulin granules to lysosomes can be achieved by several mechanisms including macroautophagy and macroautophagy-like pathways. However, our findings indicate that starvation- or metabolic stress-induced SINGD is macroautophagy-independent and even inhibits macroautophagy through mTORC1 activation.

Our current study together with our and others’ published evidence^31,32^ demonstrate that β cells tightly control the fate of newly formed cargo-containing carriers. On the cellular level, this means that SGs on their route to the plasma membrane need to bypass the lysosomes in the Golgi area. In light of the well-known mechanisms of protein quality control in endoplasmic reticulum (ERAD), it is tempting to speculate that the SINGD pathway might play a similar role at the Golgi: SGs incapable of interacting with the secretory trafficking machinery, or containing wrong cargos, etc. might need to be efficiently cleared to prevent ‘clogging’ of the Golgi area with non-functional byproducts of secretion. Our data indicate that SINGD is relatively low under basal conditions, and it is markedly enhanced upon nutrient stress, leading to depletion of SGs. The comparison between SGs targeted for degradation and SGs involved in secretion remains to be done. Nonetheless, in line with this hypothesis, enhanced degradation of SGs was previously observed in models of perturbed insulin release, and was therefore considered as a means to counter-balance SG accumulation^28,92^. Importantly, however, our study suggests that aberrant SINGD might be directly involved in the pathogenesis of T2D: first, we observed an exacerbation of T2D upon triggering SINGD and second, preventing SINGD by keeping PKD active or by reducing the levels of CD63 restored the SG pool and insulin secretion.

Altogether, our findings demonstrate that, under diabetic conditions, stress-induced nascent granule degradation (SINGD) interferes with the protective role of macroautophagy in β cells, which may contribute to premature β cell failure in T2D. Our study also links lysosomal degradation of SGs to β cell failure and provides the first steps towards understanding the molecular mechanisms of the SINGD pathway, suggesting potential therapeutic avenues in T2D.

## Methods

### Common reagents

Reagents were obtained from the following sources: HRP-conjugated anti-mouse and anti-rabbit secondary antibodies from IGBMC; oligonucleotides, type V Collagenase solution, Bafilomycin A1 from Sigma Aldrich; Mowiol from Calbiochem; cOmplete Protease inhibitor cocktail tablets from Roche; Rapamycin and CID755673 from Tocris Biosciences; 4′,6-diamidino-2-phenylindole (DAPI), Image-iT® FX signal enhancer and Alexa −488, −568, and −647 conjugated secondary antibodies from Life Technologies; Osmotic pumps from Alzet®. Golgi-CFP and GFP-CD63 were obtained from Addgene. Golgi-CFP was a gift from Alexandra Newton, University of California San Diego (Addgene plasmid # 14873^93^). It contains the sequence encoding the amino-terminal 33 residues of Golgi-associated endothelial nitric-oxide synthase in frame with the 5′-end of CFP. GFP-CD63 (CD63-pEGFP C2) was a gift from Paul Luzio (Addgene plasmid # 62964). It contains full-length human CD63 cloned into pEGFP C2.

### Primary antibodies used

Fig. 1a: insulin, Cell Signaling Technology (CST), produced in rabbit (4590) (1/100). Giantin G1/133, Enzo life science monoclonal, mouse, ALX-804-600-C100) (1/1000).

Fig. 1b: insulin, CST, produced in rabbit (4590) (1/100). Anti rat-CD63 produced in mouse Biorad (AD-1), formerly Serotec (1/100).

Fig. 2a and Supplementary Movie 2: anti rat-CD63 produced in mouse Biorad (AD-1), formerly Serotec (1/100). mTOR, 7C10, CST, produced in rabbit (2983) (1/200).

Fig. 2b: phospho-ULK1 (Ser757), CST produced in rabbit (6888).

Fig. 2d: LC3B antibodies: (1) 2G6, produced in mouse, Nanotools (1/1000) and (2) Novus, produced in rabbit, (NB100-2220) (1/1000). GAPDH, Sigma, produced in rabbit (G9545) (1/10,000).

Fig. 3a: insulin, CST, produced in mouse L6B10 (8138) (1/100). CD63, produced in rabbit, from P. Saftig (1/100).

Fig. 3e: insulin, CST, produced in rabbit 4590 (1/100). p62, Progen, produced in guinea pig (GP62-C) (1/100).

Fig. 3f: p62, progen, produced in guinea pig (GP62-C) (1/1000). GAPDH Sigma produced in rabbit (G9545) (1/10,000).

Fig. 4a: anti-phospho-PRKD1 (pSer910), Sigma, produced in rabbit (SAB4300075) (1/1000). Tubulin, Sigma, produced in mouse (T9026) (1/10,000). Insulin, Sigma, produced in guinea Pig (I8510).

Fig. 4c: PKD/PKCμ Antibody, CST, rabbit (2052) (1/1000). Tubulin, Sigma, produced in mouse (T9026) (1/10,000).

Fig. 4f: anti-phospho-PRKD1 (pSer910), Sigma, produced in rabbit (SAB4300075) (1/1000). PKD/PKCμ Antibody, CST, rabbit (2052) (1/1000). GAPDH Sigma produced in Rabbit (G9545) (1/10,000).

Fig. 4g, h: insulin, Sigma, produced in Guinea Pig (I8510). GAPDH Sigma produced in Rabbit (G9545) (1/10,000).

Fig. 5a: insulin, CST, produced in Rabbit (4590) (1/100).

Fig. 5b: insulin, Sigma, produced in mouse K36AC10 (I2018) (1/1000).

Fig. 5c: C-peptide I, produced in mouse, Biorad, Formerly Serotec (MCA2857) (1/1000). GAPDH Sigma produced in Rabbit (G9545) (1/10,000).

Fig. 5d: insulin, CST, produced in Rabbit (4590) (1/100). Anti rat-CD63 produced in Mouse Biorad (AD-1), formerly Serotec (1/100).

Fig. 5e: insulin, Sigma, produced in Guinea Pig (I8510). GAPDH Sigma produced in Rabbit (G9545) (1/10,000).

Supplementary Fig. 1a: insulin, Sigma, produced in mouse K36AC10 (I2018) (1/1000). Insulin, CST, produced in Rabbit (4590) (1/100)

Supplementary Fig. 1b: insulin, CST, produced in Mouse L6B10 (8138) (1/100). Lamp2, Invitrogen, produced in Rabbit (1/700).

Supplementary Fig. 2c: GFP, ThermoFisher Scientific, produced in rabbit, A6455. GAPDH, Sigma, produced in Rabbit (G9545) (1/10,000).

Supplementary Fig. 2e: insulin, CST, produced in Rabbit (4590) (1/100).

Supplementary Fig. 2i: anti rat-CD63 produced in Mouse Biorad (AD-1), formerly Serotec (1/100).

Supplementary Fig. 4a: insulin, CST, produced in Rabbit (4590) (1/100).

Supplementary Fig. 4b: an antibody against Phogrin was a generous gift from John Hutton and Howard Davidson (University of Colorado) (1/1000).

Supplementary Fig. 4c: Beclin-1, CST, produced in rabbit (3495) (1/1000). ATG5, CST, produced in rabbit (12994) (1/1000). GAPDH Sigma produced in Rabbit (G9545) (1/10,000).

Supplementary Fig. 4i: LC3B antibody (PM036) from MBL.

Supplementary Fig. 7d: insulin, CST, produced in Mouse L6B10 (8138) (1/100). CD63, produced in Rabbit, from P. Saftig (1/100).

### Mice

BTBR *ob/+* ^42^ (BTBR background) mice were obtained from Charles River and were maintained by heterozygous breeding to generate +/*+* and *ob/ob* littermates. *Cd63*^−/−^ mice (C57BL/6 × 129/SV background) were generated and described previously^50^. Mice were housed under controlled temperature on a 12-h light/dark cycle with unrestricted access to water and standard laboratory chow. Maintenance and animal experimentation were in accordance with the local ethical committee (Com’Eth) in compliance with the European legislation on care and use of laboratory animals.

### Mice experiments

Male mice were used in all experiments. For the High Fat Diet (HFD) experiments, mice (C57BL/6 × 129/SV background) were put under HFD (d12492i from Research diet®) for 13 weeks starting at 5 weeks of age and were monitored weekly. For pharmacological treatments, mice were implanted with osmotic pumps according to the manufacturer protocol at 4 weeks of age under anesthesia induced by 3–4% Isoflurane inhalation and maintained with 1.5% Isoflurane. CID755676 was dissolved at 10 mg/ml in 50% DMSO solution. Compound A was diluted in DMSO. For Glucose Stimulated Insulin Secretion (GSIS) test, the mice were fasted and intraperitoneally injected with a glucose solution (2 g/kg body weight). 100 µl blood samples were repeatedly taken from the tail tip. Samples were immediately centrifuged and plasma was stored at −20 °C. Insulin was measured in 25 µl plasma using Insulin Ultrasensitive ELISA (ALPCO).

### Molecular cloning and INS^PGCD^ knock-in cell line generation

The modified repair plasmid was generated by amplifying the rat CD63 C-terminal sequence with modified primers complementary for the DsRed sequence. The DsRed sequence was amplified from a DsRed containing plasmid with modified primers complementary for the CD63 C-terminal region. 3 PCR fragments were put together with a SmaI digested opened puc57 plasmid and treated with ExoIII nuclease which selectively removes the 3′ end of double stranded DNA fragment and allow complementary annealing between the PCR fragments and the puc57 plasmid. The recombinant plasmid was transformed and amplified in STBL3 competent *E.coli* and verified by Sanger sequencing (GATC). The same strategy was used to generate the Phogrin-eGFP mutants (PTPRN2-eGFP).

Primers sequence (5′−3′) of puc57-CD63 HR1 Fw:

CGAATGCATCTAGATATGGGATCCTGACTGGGCCAGAGCAAGCCCTTTAAAT; CD63 HR1-DsRed Rv:

GTCCTCGGTGTTGTCCATTACTTCGTAGCCACTCCGGATAC;

CD63 HR1-DsRed Fw:

GAGTGGCTACGAAGTAATGGACAACACCGAGGACGTCATCAAGG;

DsRed-CD63 HR2 Rv:

TCAGACACGCCCCCACCTCACTGGGAGCCGGAGTGGC;

DsRed-CD63 HR2 Fw:

CCACTCCGGCTCCAGTGAGGTGGGGGCGTGTCTGAGCTC;

CD63 HR2-puc57 Rv:

GCCTCTGCAGTCGACGGGCCCGGGCTTTACAGCAAGAGGCTGGTTTCGG; puc57-PTPRN2 HR1 Fw:

CGAATGCATCTAGATATCGGATCCAGGCAGGGGCTGGTTCTGATTGCAC; PTPRN2 HR1-eGFP Rv:

TGAACAGCTCCTCGCCCTTGCTCACCTGGGGAAGGGCCTTCAGGATGGCA; PTPRN2 HR1-eGFP Fw:

TGCCATCCTGAAGGCCCTTCCCCAGGTGAGCAAGGGCGAGGAGCTGTTCA; eGFP-PTPRN2 HR2 Rv:

TCCCGTCAGCTCCAGCTTCGGTGCCTACTTGTACAGCTCGTCCATGCCGA; eGFP-PTPRN2 HR2 Fw:

TCGGCATGGACGAGCTGTACAAGTAGGCACCGAAGCTGGAGCTGACGGGA PTPRN2 HR2-puc57 Rv:

GCCTCTGCAGTCGACGGGCCCGGGCAAACTGGAGCATCCAGTGAGAATGT. The specific gRNAs were designed (crispr.mit.edu) and purchased at Sigma Aldrich. They were cloned inside the Cas9-containing pX330 plasmid according to the manufacturer protocols (Addgene). Additional nucleotides for correct insertion after BbsI digestion were inserted.

gRNA sequences (5′−3′) of CD63 KI gRNA 1 Fw:

caccGGGCTACGAAGTAATGTAGGGTGG;

CD63 KI gRNA 1 Rv:

aaacCCACCCTACATTACTTCGTAGCCC;

Phogrin KI gRNA 5 Fw:

caccGTCCCCAGTAGGCACCGAAGC;

Phogrin KI gRNA 5 Rv:

aaacGCTTCGGTGCCTACTGGGGAC.

To generate knock-in cell line, INS1 cells were transfected with corresponding plasmids (20 µg each). To select single clones, cells were diluted at 70 cells /10 ml concentration and spread over 96-well plates, with 100 µl of media per well. Clones were screened by PCR.

Primers sequence (5′−3′) of Sequencing CD63-Dsred Fw:

CGAATGCATCTAGATATCGGATCCTGTGGTCATCATTGCAGTGGGT; Sequencing CD63-DsRed Rv:

TCTGCAGTCGACGGGCCCGGGGTACAAGGACAACATGCTCAACGAC; Sequencing Phogrin-GFP Fw:

CGAATGCATCTAGATATCGGATCCTCAACATAAAGAGCAGAGGCCAAC;

Sequencing Phogrin-GFP Rv:

GCCTCTGCAGTCGACGGGCCCGGGCAGGTGGTAAGCCCAACGCCCAAA.

Clones positive for the insertion were then checked by Sanger sequencing (GATC), immunofluorescence analysis and/or immunoblot.

### Cell lines and transfections

INS1 cells were maintained in RPMI-1640 supplemented with 10% FCS, 10 mM Hepes, 1 mM PyrNa, 2 mM glutamine, 50 µM β-mercaptoethanol, penicillin, and streptomycin. Cells were transfected by using Amaxa Nucleofector (Lonza) according to the manufacturer’s protocol using the T-027 program.

### Islet isolation and culture

Mouse islets were isolated by type V collagenase (Sigma Aldrich) digestion and Histopaque density gradient centrifugation and allowed to recover overnight in RPMI-1640 medium 11, 1 mM glucose, supplemented with 10% (vol/vol) FBS, 10 mM Hepes, 1 mM PyrNa, 2 mM glutamine, 50 µM β-mercaptoethanol, and Penicillin/streptomycin mix. Isolated islets from non-diabetic human cadaveric donors (authorization number for the human tissue protocol: PFS12–013) were provided by the Integrated Islets European consortium for islet transplantation and the Centre Européen d’étude du diabète (CEED). Human islets were cultured in CMRL medium.

### Insulin static incubations

For static incubations, batches of 10 islets each were first kept in KRB solution containing 0.1% (wt/vol.) BSA and 2.8 mM glucose for 40 min (2 incubations 20 min each) at 37 °C, then incubated for 1 h in the presence of 2.8 or 16.7 mM of glucose. Each condition was run in triplicate. Intracellular insulin content was measured after acid–alcohol extraction. Insulin levels were measured by ELISA (ALPCO, Eurobio, Courtaboeuf, France).

### Lysates preparation and immunoblotting

Tissues or cells were rinsed once with ice-cold PBS and lysed in ice-cold lysis buffer (50 mM Tris (pH 7.5), 50 mM NaCl, 0.5% Triton X-100, 0.5% NP40 substitute, 5 mM EGTA, 5 mM EDTA, 20 mM NaF, 25 mM β-glycerophosphate, 1 mM PMSF, 0.1 mM NaVO3, 1x cOmplete protease inhibitor (Roche)). The soluble fractions of cell lysates were isolated by centrifugation at 14,000 × *g* for 10 min. Protein samples were separated by using SDS-PAGE or TRICINE-SDS-PAGE and transferred to PVDF membranes. Membranes were blocked at room temperature for 1 h with 5% skimmed milk in TBST (0.05% Tween20). Primary antibody incubation was done overnight at 4 °C. The membranes were washed three times with TBST and incubated with the secondary antibody for 1 h and finally washed again three times with TBST. Proteins were visualized with Luminata Forte ECL (Millipore) on the AI600 camera (GE health) or using x-ray films. Uncropped immunoblots are shown in Supplementary Fig. 14.

### qPCR analysis

Tissues or cells were homogenized in TRIzol reagent (Sigma). After phase separation by centrifugation, the aqueous phase was transferred into a new tube and RNA was precipitated by 75% ethanol. cDNA was synthesized with Oligo dT primers using SuperScript III forst- strand cDNA synthesis kit (Invitrogen) according to manufacturer’s protocol. Quantitative real-time PCR was performed using SYBR Green (Roche) on a LightCycler 480 (Roche). The expression values were individually normalized to housekeeping genes HPRT and GAPDH.

Primers sequence (5′−3′) of HPRT mouse fw:

AGTCCCAGCGTCGTGATTAG;

HPRT mouse rv:

TTTCCAAATCCTCGGCATAATGA;

GAPDH mouse fw: TTGGCCGTATTGGGCGCCTG;

GAPDH mouse rv: CACCCTTCAAGTGGGCCCCG;

PrKD1 mouse fw: CGTGGTTAACCCATCAAGTT;

PrKD1 mouse rv: AATTCCAAACTGTCCGGAAC;

PrKD2 mouse fw: GCAACTAGCCTGTTCTATCGTG;

PrKD2 mouse rv: AGAGCAGGATCTTGTCGTACA;

PrKD3 mouse fw: CAGTGGAAGACTTCCAGATCC;

PrKD3 mouse rv: GAATCTTGAAGGCACATCGTTTATG.

P62 mouse fw: GCTGCCCTATACCCACATCT

P62 mouse rv: CGCCTTCATCCGAGAAAC

### Immunofluorescence microscopy

INS1 cells were plated on 9–15 mm glass coverslips (Menzel-Glaser) in 24-well tissue culture plates and allowed to grow for 24 h. In the end of experiment the cells were washed once with PBS and fixed for 20 min with 4% paraformaldehyde (PFA) in PBS at room temperature. The coverslips were rinsed three times with PBS and permeabilized with 0.1% Triton X-100 in PBS for 5 min. After rinsing three times with PBS, the coverslips were incubated in Image-iT® Fx Signal enhancer for 30 min, rinsed once with PBS and incubated for 30 min in blocking buffer (3% BSA in PBS). Coverslips were subsequently incubated with primary antibodies in blocking buffer for 1 h at room temperature, rinsed three times in PBS and incubated with the secondary antibodies, diluted in blocking buffer, for 40 min at room temperature in the dark. After incubation, coverslips were rinsed three times with PBS (with DAPI added in the second washing step, when present) and mounted on glass slides using Vectashield, ProLong Gold or Mowiol and imaged with a ×100 or ×63 objective using fluorescence microscope of confocal Leica spinning Disk Andor/Yokogawa or Nikon Ti PFS.

Pancreata were fixed by intracardiac perfusion with PFA 4% in PBS. Tissues were post-fixed overnight at 4 °C in the same solution, cryoprotected in 30% sucrose in PBS solution, embedded in Cryomatrix (Thermo scientific) and kept at −80 °C. Pancreas sections were cut with a cryostat (CM3050, Leica) and mounted on Superfrost Ultra Plus slides (Menzel-Glaser). Cryosections (10 µm) were incubated for 2 h at room temperature in the blocking solution (PBS/5% Normal Goat serum/0.3% Triton X-100), then left overnight at 4 °C, in a wet chamber, with primary antibodies. After 3 washes in PBS, the sections were blocked for 15 min at room temperature in the blocking solution and incubated for 2 h at room temperature, in wet chamber, with secondary antibodies diluted in blocking solution. After three washes in PBS, one wash in milliQ water, the slides were dried and mounted in Mowiol (Calbiochem) containing DAPI (0.5 µM). Images were taken using confocal microscope Leica Spinning Disk Andor/Yokogawa or Nikon Ti PFS.

### Live microscopy

INS^PGCD^ were cultured overnight on 60µ-Dish 35 mm, high Glass bottom from Ibidi®, processed as described in the text and imaged with the Nikon Ti PFS Spinning Disk.

### Co-localization analysis

To assess pixel co-localization, we used: (a) The automatic threshold Pearson’s analysis with the Fiji plugin Co-localization thresholds. The plugin generated the co-localized pixel maps, a scatter plot as well as the Pearson’s correlation coefficient; (b) The JACOP plugin (Fiji) to determine Manders coefficients.

### Transmission electron microscopy

For transmission electron microscopy (TEM), cells and primary islets were fixed 1 h with 2.5% glutaraldehyde and 2.5% formaldehyde in 0.1 M cacodylate buffer, rinsed in buffer and followed by 1 h postfixation in 1% osmium tetroxide [OsO4] reduced by 1% potassium ferricyanure [K3Fe(CN)6] in dark on ice. After extensive rinses in distilled water, samples were then stained first by in 1% tannic acid, followed after rinses by 1% uranyl acetate, for 1 h on ice each, and rinsed in water. Samples were dehydrated with increasing concentrations of ethanol (50%, 70%, 90%, and 3 × 100%), and embedded with a graded series of epoxy resin. The blocs were finally polymerized at 60 °C for 48 h. Ultrathin sections (50 nm) were picked up on 1% pioloform coated copper slot grids and observed with a Philips CM12 operated at 80 kV equipped with an Orius SC100 CDD camera (Gatan, Pleasanton, USA).

Quantitative analysis of granule-containing lysosomes, lysosomes, and insulin granules of INS1 cells and primary islets has been assessed by TEM as in Goginashvili et al.^32^. In brief, the areas of cytoplasm and Golgi were evaluated by stereological approach using ×5600 magnification with ImageJ-based open source Fiji software package. The numbers of compartments were quantified using ×15,000 or ×19,500 magnification. Data from three independent experiments were expressed as mean±SEM. All quantitative analyses relied on systematic uniform random sampling.

### CLEM

Correlative Light and Electron Microscopy (CLEM) was performed as in Lenormand et al.^94^. In brief, INS1 cells were cultured on laser micro-patterned Aclar supports. In the end of experiment, the cells were fixed in PFA 4%, Glutaraldehyde 0.5% in 0.1 M sodium cacodylate buffer 0.1 M for 20 min at room temperature, followed by incubation in sodium cacodylate 0.1 M. Cells of interest were selected, precisely located and imaged by fluorescence confocal microscopy using Leica Spinning Disk Andor/Yokogawa microscope. Samples were then processed exactly as for TEM.

### Live-CLEM

INS1 cells were cultured on gridded coverslips (MatTek P35G-1.5-14-C-GRID). Live-cell imaging was performed using Inverted Nikon Eclipse Ti microscope (Nikon Ti PFS Spinning Disk) equipped with x100 TIRF objective (NA = 1.49), with the PFS (perfect focus system), an EMCCD camera (Evolve, Photometrics) and Yokogawa CSU-X1 Confocal Scanner. GFP and m-Cherry were excited with a 488-nm and a 561-nm laser, respectively (100 mW). Live INS1 cells were imaged at 20 frames per second. 4D imaging was performed using consecutive xy(c)tz time-lapse acquisitions. Time series of 2D sections were analyzed using FIJI and IMARIS software. In the end of experiment, the cells were immediately fixed in PFA 2.5%, Glutaraldehyde 2.5% in sodium cacodylate 0.1 M for 20 min at room temperature. The samples were then post fixed in 1% osmium tetroxide in 0.1 M cacodylate buffer for 1 h at 4 °C, dehydrated through graded alcohol (50, 70, 90, and 100%), embedded in Epon 812 cut at 70 nm (Leica Ultracut UCT) and contrasted with uranyl acetate and lead citrate. Serial sections were examined using a Philips CM12 transmission electron microscope (CM12, Philips; FEI Electron Optics, Eindhoven, the Netherlands) operated at 80 kV and equipped with an Orius 1000 CCD camera (Gatan, Pleasanton, USA).

### On-section CLEM

High accuracy on-section CLEM was performed as previously described^95,96^, with minor modifications. Cells grown on sapphire disks (Engineering Office M. Wohlwend GmbH, Sennwald, Switzerland) were high pressure frozen with a BAL-TEC HPM 010 machine. Freeze substitution (FS) and resin infiltration were performed in a Leica AFS2 machine. Cells monolayers were freeze substituted with 0.1% uranyl acetate in acetone for 9 h at −90 °C. The temperature was then increased (5 °C/h) to −45 °C and samples were incubated for 5 h. The FS solution was then washed out with dry acetone and samples were infiltrated with Lowicryl HM20 (Polyscience Europe GmbH, Eppelheim, Germany) at increasing concentration (10%, 25%, 50%, 75%, 3 × 100%), while the temperature was increased to −25 °C. The resin was then polymerized under UV light for 48 h. In all, 300-nm-thick sections were cut from the polymerized resin block and picked up on carbon coated mesh grids. TetraSpeck fluorescent microspheres (100 nm) (Lifetechnologies) were adsorbed to the grid for the subsequent fiducial-based correlation between light (LM) and electron microscopy (EM) images. Fluorescence imaging of the sections was performed as in Kukulski et al.^95^ using an Olympus ScanR microscope and a 100 × 1.4NA oil objective. Tilt series of the areas with fluorescent structures of interest were acquired with a FEI Tecnai F30 electron microscope. Tomograms were then reconstructed using the IMOD software package^97^. Correlation of LM and EM images was performed by manually assigning the position of tetraspecks in the two imaging modalities using ec-CLEM plugin^98^ from ICY software^99^.

### FIB-SEM

Freshly isolated pancreatic islets were fixed with 2.5% Glutaraldehyde (Electron Microscopy Sciences), 0.1% Malachite green oxalate (Sigma) in 0.1 M PHEM buffer. Subsequently, islets were washed with 0.1 M PHEM buffer and stored at 4 °C until further processing in a PELCO BioWave Pro microwave processor (Ted Pella, Inc.)^100^. The processing procedure was as follows: post-fixation with 1% osmium tetroxide (OsO_4_; Electron Microscopy Sciences) + 0.8% potassium hexacyanoferrate (III) (K_3_Fe(CN)_6_; Merck) in H_2_O, rinsed with H_2_O, post-stained with 1% tannic acid, rinsed with H_2_O, en bloc stained with 0.5% UA in H_2_O, rinsed with H_2_O, dehydrated with increasing concentrations of ethanol (25, 50, 75, 90, and 100%) and embedded with a graded series of Durcupan (Sigma-Aldrich). Subsequently the Durcupan infiltrated islets were incubated at 60 °C for 48 h to polymerize the resin. Once polymerized, resin embedded islets were trimmed to expose two surfaces of material. After trimming, the samples were mounted onto the edge of an SEM stub (Agar Scientific) with silver conductive epoxy (CircuitWorks) with one of the trimmed surfaces facing up so that it was perpendicular to the focused ion beam (FIB). The sample was then sputter coated with gold (180 s at 30 mA) in a Quorum Q150RS coater before being placed in the Zeiss Auriga 60 focused ion beam scanning electron microscope (FIB-SEM). Once the ROI was located in the sample, Atlas3D software (Fibics Inc. and Zeiss) was used to perform sample preparation and 3D acquisitions. First a platinum protective coat of (20 µm×20 µm) was deposited with 1 nA FIB current. A small rough trench was then milled to expose the imaging cross-section with 16 nA FIB current, followed with a polish at 4 nA. The 3D acquisition milling was done with 1 nA FIB current. For SEM imaging, the beam was operated at 1.5 kV with 60 µm aperture using High Current mode and the EsB detector (1.1 kV collector voltage) at a dwell time from 10 µs with no line averaging over a pixel size of 4 × 4 nm and slice thickness 8 nm. All acquired images were then combined as a stack with FIJI (ImageJ) and subsequently aligned using the TrakEM2 plugin. Finally, the contrast of all images was inverted to have the same contrast as that of conventional TEM images.

### Generation of Compound A selectively inhibiting MAPK p38δ

Following the screening of a set of 808 000 compounds from the Sanofi proprietary compound collection using a HTRF assay on purified p38δ with ATF2 as substrate, compounds of the pyrimido-azepinone family were identified as moderate inhibitors of p38δ. After a short chemical optimization cycle, Compound A (Supplementary Fig. 11a) was selected as a tool compound to study the effects of p38δ inhibition in various in vitro assays and on blood glucose levels in BTBR *ob*/*ob* mice described in this paper.

Compound A demonstrated a very good selectivity profile versus p38α and PKD1, as measured by mobility shift assays (Caliper) while it showed an equivalent inhibitory effect against p38γ (Supplementary Fig. 11b). Furthermore, Compound A exhibited a very good selectivity profile in a panel of 60 kinases (Supplementary Fig. 11c) with the exception of p70S6K (59% inhibition at 10 μM) and mTOR (48% inhibition at 10 μM). Inhibition of p38γ was also confirmed in this panel (alias SAPK3: 64% inhibition at 10 μM). A more detailed account of the discovery, chemistry and biochemical properties of this novel class of selective p38δ inhibitors will be given elsewhere.

### Statistical analysis

Sample sizes for all types of experiments in this study were chosen according to published guidelines and to our previous experience. In all imaging-based quantitative experiments, systematic uniform random sampling was used. The statistical significance of the differences between two groups was investigated by *t*-test or by Mann–Whitney *U*-test. At least three independent experiments were performed for each comparison. The statistical analysis was performed using Excel or GraphPad Prism software (GraphPad Software Inc.).

### Reporting summary

Further information on research design is available in the Nature Research Reporting Summary linked to this article.

## Supplementary information

Supplementary Information

Description of Additional Supplementary Files

Supplementary Movie 1

Supplementary Movie 2

Supplementary Movie 3

Reporting Summary

Source Data

## Data Availability

All relevant data that support the conclusions of this study are available from the authors on reasonable request (see author contributions for specific data sets). The source data underlying Figs. 1b, c, e, 2b, c, e, 3b, e, 4a–c, e, i, j, and 5b, f and Supplementary Figs. 3a–c, 4a, b, g, h, 6, 7a–c, 8a, b, 9, 10, 11d, 12a, c, d, and 13a–c are provided as a Source Data file. Uncropped immunoblots are shown in Supplementary Fig. 14. Analysis of PKD1 (*Prkcm*) expression in islets of wt and *ob*/*ob* BTBR mice^45^ has been performed at http://diabetes.wisc.edu/
