# Supplementary material for: Lysosomal degradation of newly formed insulin granules contributes to β cell failure in diabetes
Source: Nat Commun. 2019 Jul 25;10:3312. doi: 10.1038/s41467-019-11170-4 (PMC6658524; doi:10.1038/s41467-019-11170-4)
Supplement: Supplementary file 1 — Supplementary Information [file 41467_2019_11170_MOESM1_ESM.pdf]

Lysosomal degradation of newly formed insulin granules contributes to β cell failure in diabetes

Pasquier *et al*.

Supplementary information

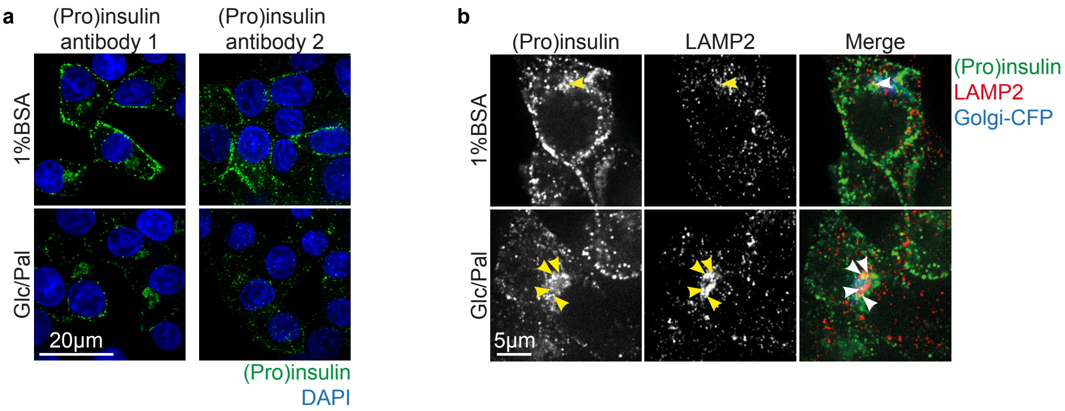

Supplementary Figure 1: Glucose/Palmitate treatment induces co-localisation of secretory granules with lysosomes in the Golgi area. (**a**) Immunofluorescence (IF) of insulin+proinsulin ("(pro)insulin") (green) in INS1 cells treated with control (1% BSA, 11 mM glucose, "1% BSA") or glucolipotoxic (1% BSA, 33.3 mM Glucose and 0.4 mM Palmitate, "Glc/Pal") media for 20h. Nuclei were stained with DAPI. (**b**) IF of (pro)insulin (green) and LAMP2 (red) in INS1 cells treated with 1% BSA or Glc/Pal media for 20h. Golgi-CFP was used to visualize the Golgi apparatus. Arrowheads point to (pro)insulin/LAMP2 co-localization.

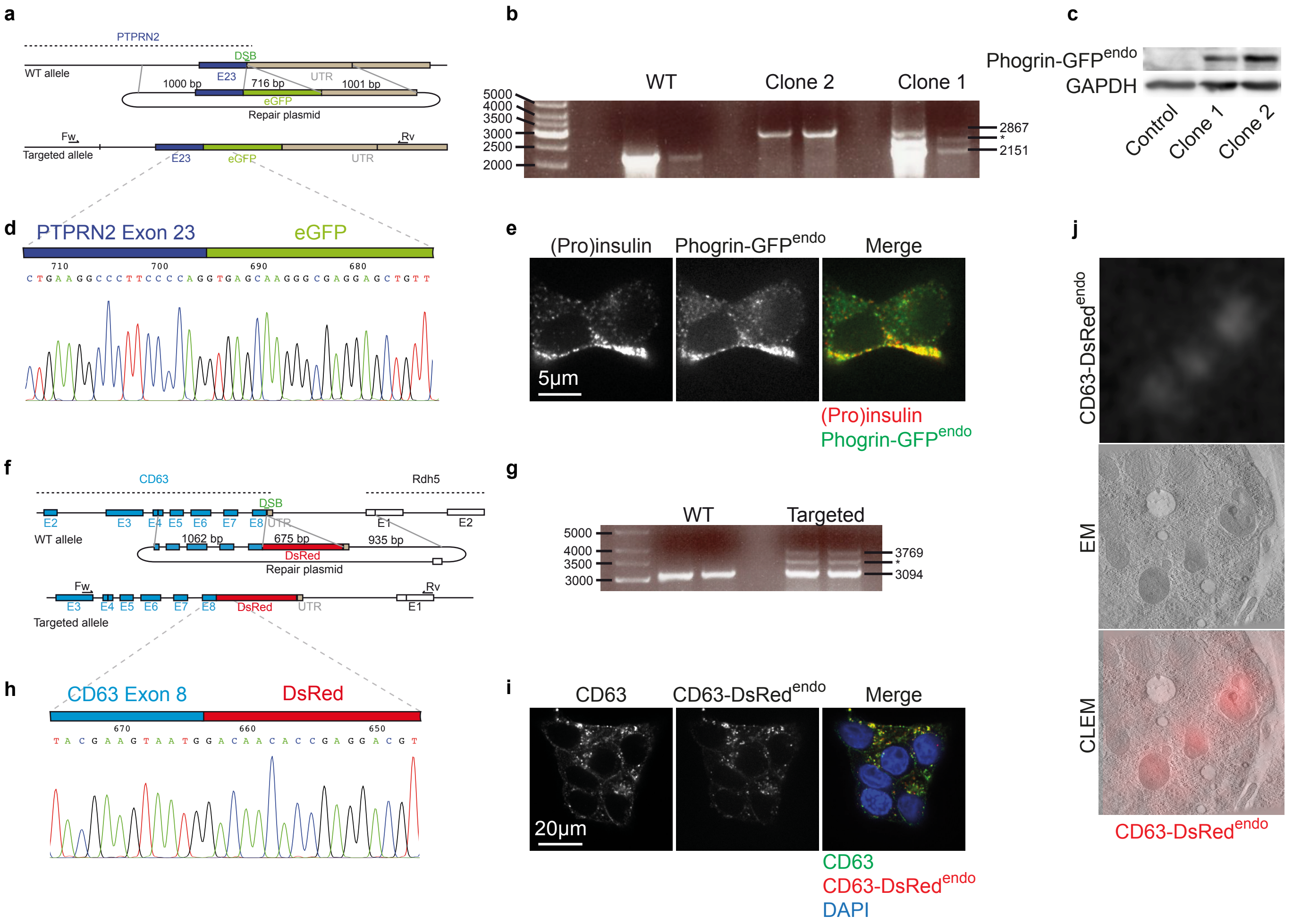

Supplementary Figure 2. Generation of the INS1PGCD knock-in cells. (**a**) Diagram showing the strategy for homologous recombination. EGFP was introduced in frame 3' of the last exon of *PTPRN2* (Phogrin). Forward and Reverse genotyping primers are designated as Fw and Rv, respectively. DSB, Double-stranded break. E, exon. UTR, Untranslated Region. (**b**) Validation of single clones by PCR. Expected sizes of targeted (2867bp) or wild type (2151bp) alleles are indicated. Clone 1 contained heterozygous insertion, clone 2 contained homozygous insertion of eGFP coding sequence. *, unspecific band. (**c**) Immunoblot of GFP using lysates from clones 1 and 2. GFP band matched the expected size of GFP-Phogrin^{endo} at 100 kDa. GAPDH was used as a loading control. Clone 2 was used to generate a double knock-in line. (**d**) Sanger sequencing verification of eGFP insertion. (**e**) Immunofluorescence (IF) of insulin+proinsulin ("(pro)insulin") (red) and Phogrin-GFP^{endo} (green) in Phogrin-GFP knock-in INS1 cells. (**f**) Diagram showing the strategy for homologous recombination. DsRed was introduced in frame 3' of the last exon of *CD63*. (**g**) Validation of single clones by PCR. Expected sizes of targeted (3769bp) or wild type (3094bp) alleles are indicated. (**h**) Sanger sequencing verification of DsRed insertion. (**i**) IF of CD63 and CD63-DsRed^{endo} in CD63-DsRed knock-in INS1 cells. (**j**) On-section Correlative Light and Electron Microscopy analysis of CD63-DsRed knock-in INS1 cells.

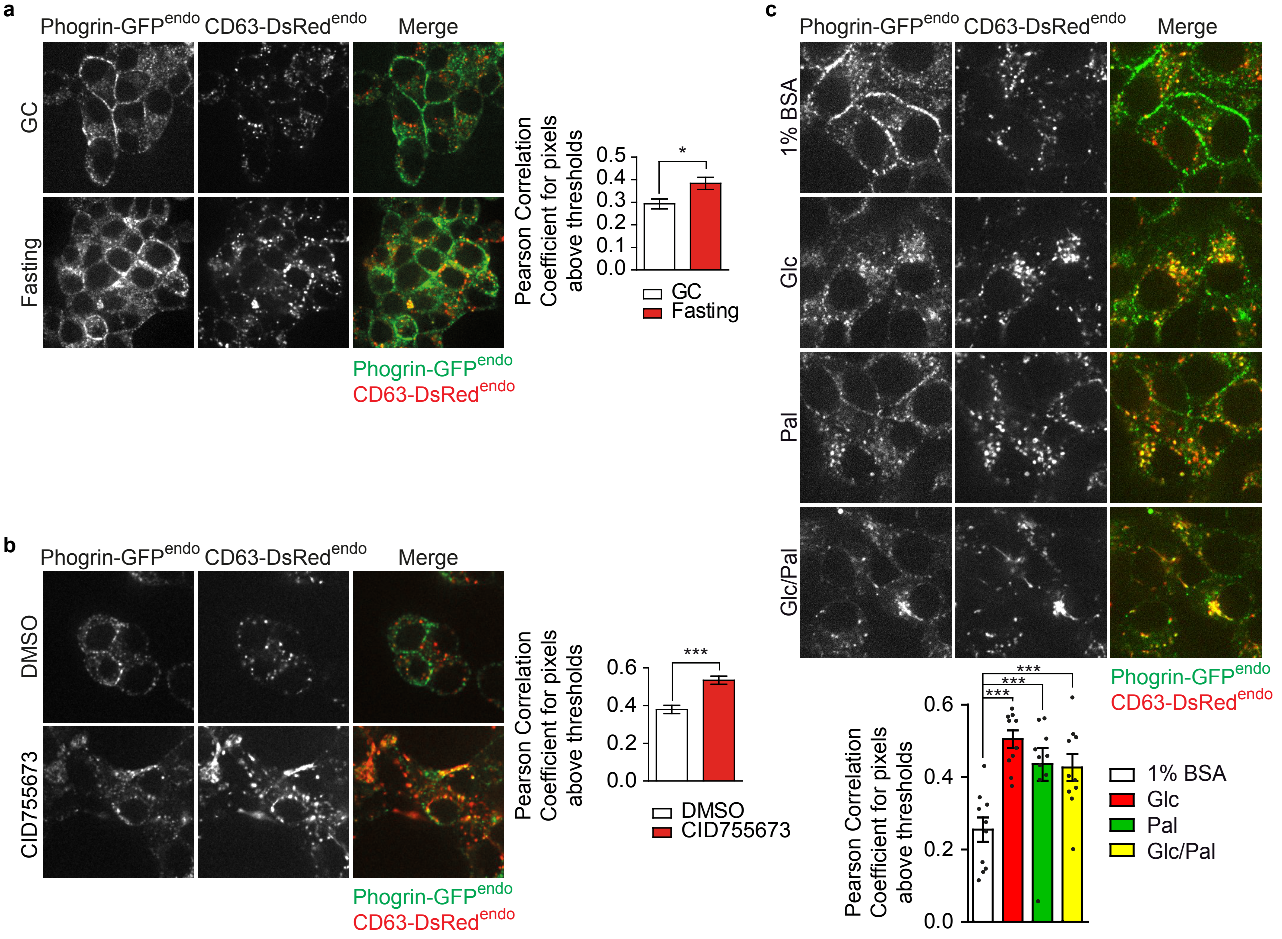

Supplementary Figure 3. Increased co-localization between Phogrin-GFP^{endo} and CD63-DsRed^{endo} upon fasting, PKD inhibition, gluco- and lipotoxic conditions in INS1^{PGCD} cells. (**a**) Live-cell imaging and quantification of the co-localization events between Phogrin-GFP^{endo} (green) and CD63-DsRed^{endo} (red) in INS1^{PGCD} cells upon growing culture (GC) or fasting (Hank's Balanced Salt Solution, 2.8 mM Glucose) conditions for 1h using Pearson's correlation coefficient for pixels above threshold. N_{GC}=26; $N_{Fasting}$=19; *P<0.05, two-tailed t-test. (**b**) Live-cell imaging and quantification of the co-localization events between Phogrin-GFP^{endo} (green) and CD63-DsRed^{endo} (red) in INS1^{PGCD} cells upon control treatment (DMSO) or PKD inhibition (CID755673, 30 µM) for 12h using Pearson's correlation coefficient for pixels above threshold. N_{DMSO}=12; $N_{CID755673}$=12; ***P<0.001, two-tailed t-test. (**c**) Live-cell imaging and quantification of the co-localization events between Phogrin-GFP^{endo} (green) and CD63-DsRed^{endo} (red) in INS1^{PGCD} cells treated with control (1% BSA, 11 mM glucose, "1% BSA"), glucotoxic (1% BSA, 33.3 mM Glucose, "Glc"), lipotoxic (1% BSA, 0.4 mM Palmitate, "Pal") or glucolipotoxic (1% BSA, 33.3 mM Glucose and 0.4 mM Palmitate, "Glc/Pal") media for 40h using Pearson's correlation coefficient for pixels above threshold. N=10 per each condition. ***P<0.001, two-tailed t-test. In **a**, **b** and **c** data are shown as mean ± SEM, source data are provided as a Source Data file.

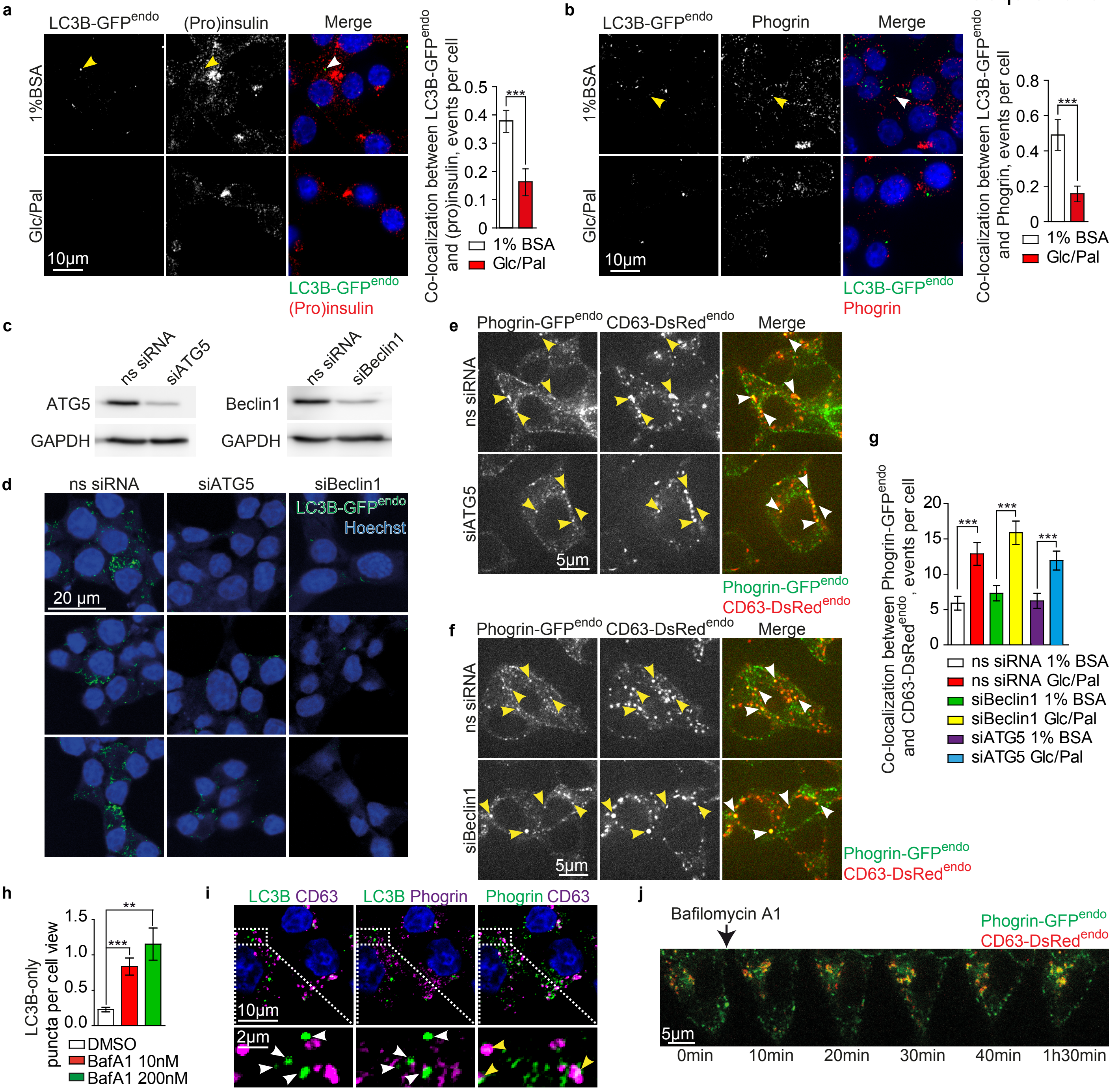

Supplementary Figure 4. Metabolic stress-induced lysosomal degradation of secretory granules is independent of macroautophagy. (**a**) Immunofluorescence (IF) of LC3B-GFP^{endo} (green) and (pro)insulin (red) in INS1$^{LC3B-GFPendo}$ cells treated with control (1% BSA, 11 mM glucose, "1% BSA") or Glc/Pal media for 48h. Arrowheads point to LC3B-GFP^{endo}/(pro)insulin co-localization. Quantification of LC3B-GFP^{endo}/(pro)insulin puncta per cell. $N_{1\%BSA}$=78; $N_{Glc/Pal}$=67; ***P<0.001, two-tailed t-test. (**b**) IF of LC3B-GFP^{endo} (green) and Phogrin (red) in INS1$^{LC3B-GFPendo}$ cells treated as in **a**. Arrowheads point to LC3B-GFP^{endo}/phogrin co-localization. Quantification of LC3B-GFP^{endo}/phogrin puncta per cell. $N_{1\%BSA}$=109; $N_{Glc/Pal}$=56; ***P<0.001, two-tailed t-test. (**c**) Immunoblot of indicated proteins using lysates of INS1^{PGCD} cells transfected with indicated siRNAs and treated with Glc/Pal media for 48h following transfection. GAPDH was used as a loading control. (**d**) IF of LC3B-GFP^{endo} (green) in INS1$^{LC3B-GFPendo}$ cells transfected with indicated siRNAs for 48h and treated with Bafilomycin A1 (BafA1), 10nM for the last 2h of incubation. (**e, f**) Imaging of INS1^{PGCD} cells endogenously expressing Phogrin-GFP^{endo} (green) and CD63-DsRed^{endo} (red) transfected with indicated siRNAs and treated with Glc/Pal media for 48h following transfection. Arrowheads point to Phogrin-GFP^{endo}/CD63-DsRed^{endo} co-localization. (**g**) Quantification of co-localization events per cell between Phogrin-GFP^{endo} (green) and CD63-DsRed^{endo} for experiments shown in **e** and **f**. $N_{ns\ siRNA\ BSA}$=30; $N_{ns\ siRNA\ Glc/Pal}$=38; $N_{siBeclin1\ BSA}$=49; $N_{siBeclin1\ Glc/Pal}$=31; $N_{siATG5\ BSA}$=54; $N_{siATG5\ Glc/Pal}$=39. ***P<0.001, two-tailed t-test. (**h**) Quantification of LC3B-only (LC3B-positive/CD63-negative) puncta corresponding to autophagosomes in INS1^{PGCD} cells treated with Glc/Pal media for 48h, and treated for the last 4h of incubation as indicated. N_{DMSO}=352; $N_{BafA1\ 10nM}$=134; $N_{BafA1\ 200nM}$=160. **P<0.01; ***P<0.001; one-tailed, unpaired t-test with Welch's correction. (**i**) IF of LC3B, Phogrin-GFP^{endo} and CD63-DsRed^{endo} in INS1^{PGCD} cells treated with Glc/Pal media for 48h and treated with BafA1, 10nM for the last 4h. White arrowheads point to accumulated LC3B-positive/CD63-negative autophagosomes, negative for Phogrin. Yellow arrowheads point to Phogrin-positive/CD63-positive granule-containing lysosomes. (**j**) Live-cell imaging of INS1^{PGCD} cells treated with BafA1, 15nM for indicated times. Cells were pre-treated with Glc/Pal media for 48h. In **a**, **b**, **g**, and **h** data are shown as mean ± SEM, source data are provided as a Source Data file.

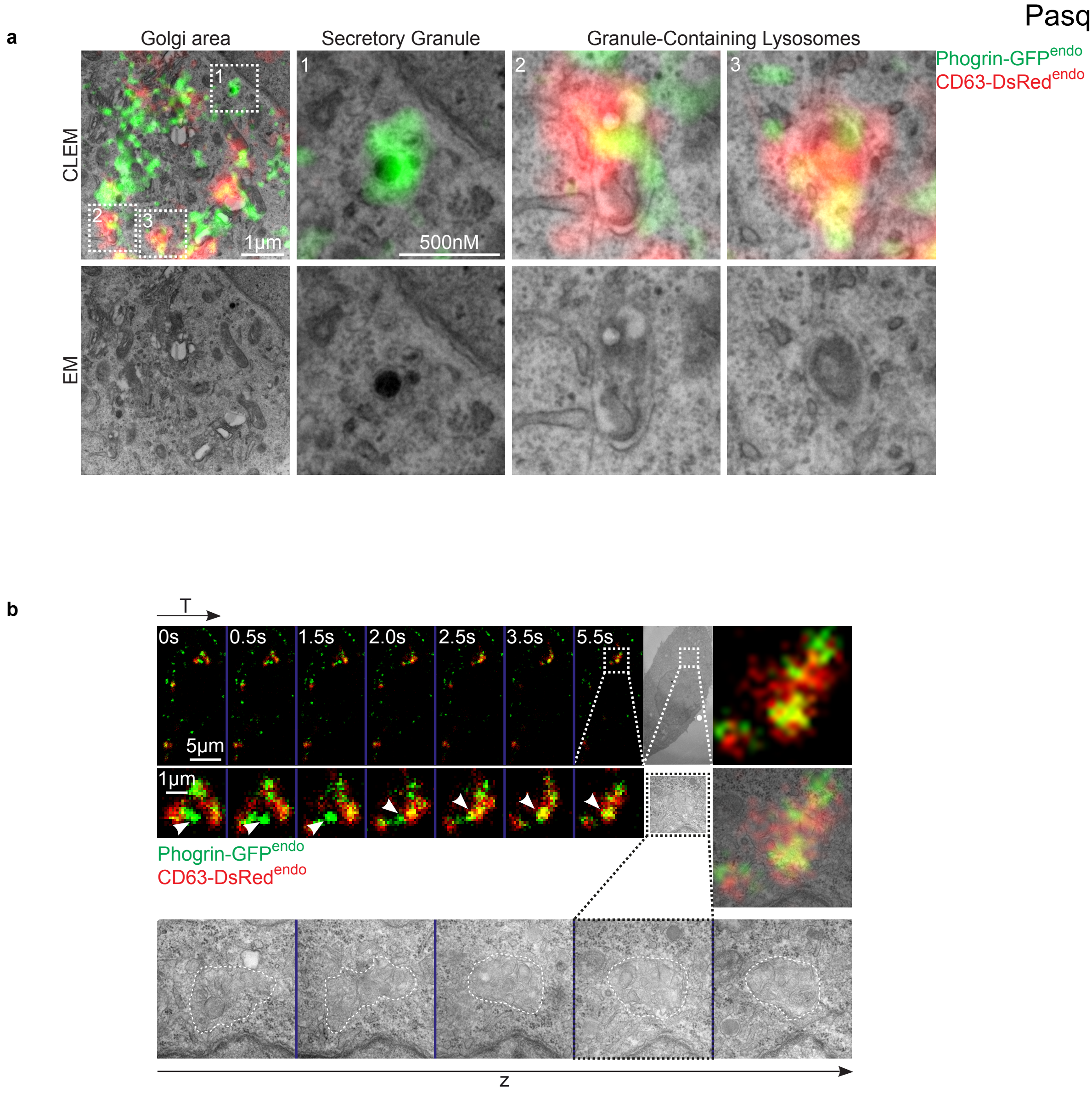

Supplementary Figure 5. Correlative light and electron microscopy (CLEM) analysis of INS1^{PGCD} cells endogenously expressing Phogrin-GFP^{endo} and CD63-DsRed^{endo}. (**a**) CLEM of INS1^{PGCD} cells treated with glucolipotoxic (1% BSA, 33.3 mM Glucose and 0.4 mM Palmitate, “Glc/Pal”) media for 20h. Insets show a Phogrin-GFP^{endo}-positive secretory granule and Phogrin-GFP^{endo}/CD63-DsRed^{endo}-positive granule-containing lysosomes. (**b**) Top: Live cell imaging of INS1^{PGCD} cells treated with Glc/Pal for 24h and imaged at indicated times, which was followed by CLEM (live-CLEM). Arrowheads point to a fusion event between secretory granule and granule-containing lysosome. Live video is shown in **Supplementary Movie 1**. Bottom: serial EM sections of the region of interest.

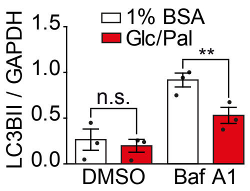

LC3BII / GAPDH:

DMSO	1% BSA=0.09	
	Glc/Pal=0.11	
Baf A1	1% BSA=0.92	
	Glc/Pal=0.64	

Supplementary Figure 6. Autophagic flux in β cells is decreased upon glucolipotoxic conditions. Quantification of LC3BII/GAPDH ratio from immunoblots shown in **Fig. 2d**. for INS1 cells (left, N=3 per each group; n.s.: not significant, **P<0.01, two-tailed t-test) and human islets (right, N=1), source data are provided as a Source Data file.

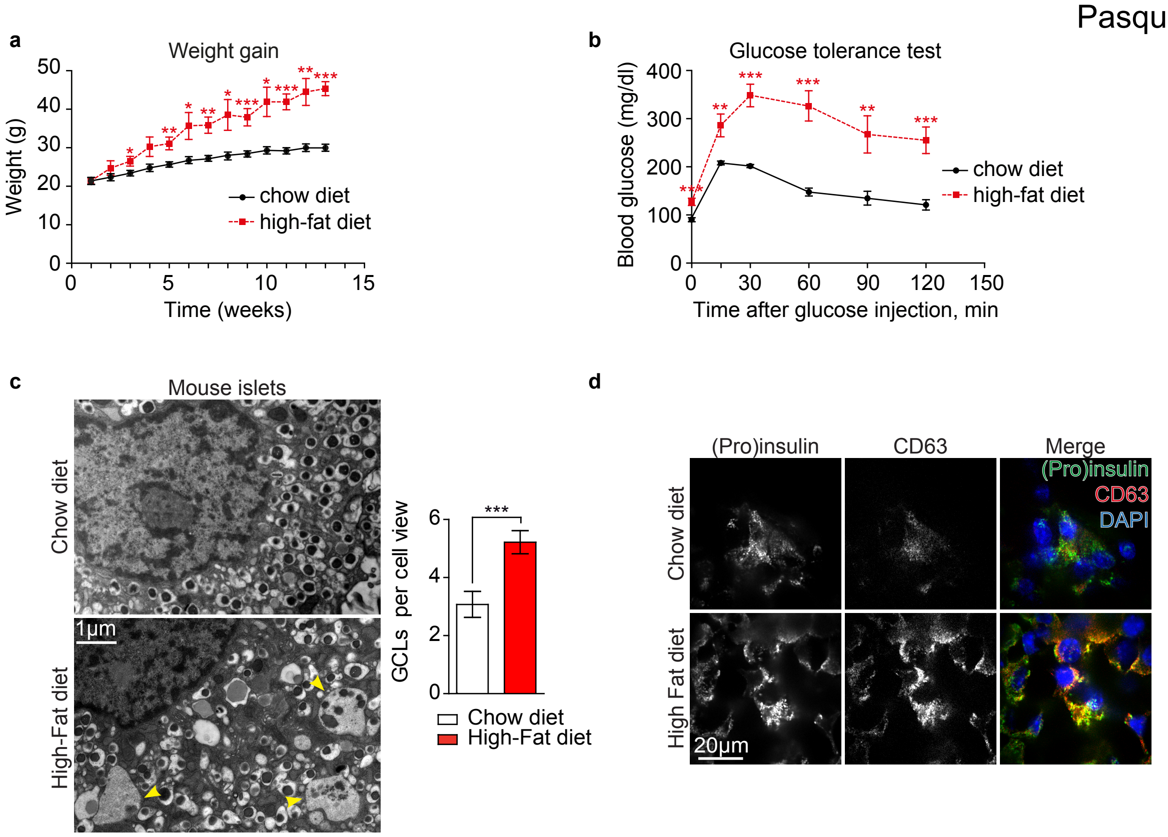

Supplementary Figure 7. Increased granule-containing lysosomes (GCLs) in β cells of High Fat diet-fed mice. (**a**) Time-course of the body weight of chow diet-fed mice or high fat diet-fed mice. N=6-11 mice per time point; *P<0.05, **P<0.01, ***P<0.001, two-tailed t-test.(**b**) Glucose tolerance test (GTT) in mice fed with chow diet or high fat diet for 13 weeks. N=11 mice per group; **P<0.01, ***P<0.001, two-tailed t-test. (**c**) Electron microscopy analysis of β cells of mice islets from mice fed with chow diet or high fat diet for 13 weeks. Arrowheads indicate GCLs. Quantification of granule-containing lysosomes (GCLs) per cell view. N_{CD}=27; N_{HFD}=37 from 3 mice per group; ***P<0.001, two-tailed t-test.(**d**) Immunofluorescence (IF) of (pro)insulin (green) and CD63 (red) in β cells of mice fed with chow diet or high fat diet for 13 weeks. N=3 mice per group. In **a**, **b** and **c** data are shown as mean ± SEM, source data are provided as a Source Data file.

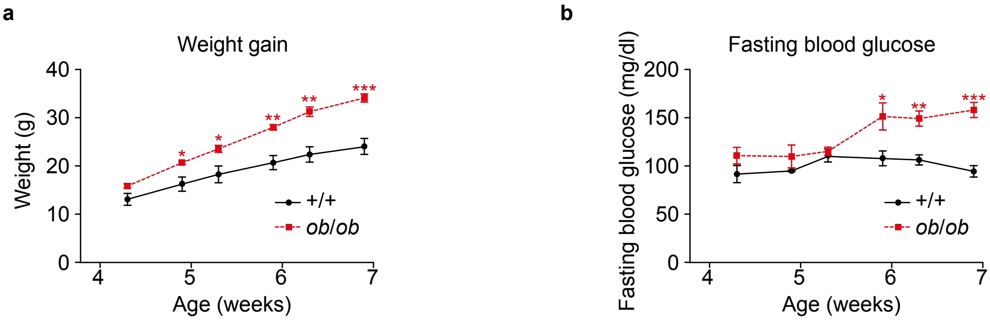

Supplementary Figure 8. BTBR *ob*/*ob* mice progressively develop diabetes at early age. (**a**)Time-course of the body weight of BTBR +/+ and BTBR *ob*/*ob* mice. $N_{+/+}$=5; $N_{ob/ob}$=5; *P<0.05, **P<0.01, ***P<0.001, two-tailed t-test. (**b**) Time-course of fasting blood glucose levels of BTBR +/+ and BTBR *ob*/*ob* mice. $N_{+/+}$=5; $N_{ob/ob}$=5; *P<0.05, **P<0.01, ***P<0.001, two-tailed t-test. In **a** and **b** data are shown as mean ± SEM, source data are provided as a Source Data file.

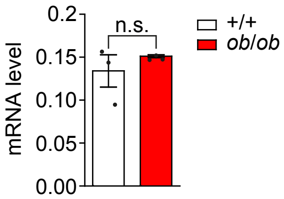

Supplementary Figure 9. p62 mRNA levels are similar in BTBR +/+ and BTBR *ob*/*ob* mice. Quantitative RT-PCR using mRNA isolated from pancreatic islets of 8-week old BTBR +/+ and BTBR *ob*/*ob* mice using primer pairs amplifying cDNA of p62.The expression values were normalized to a housekeeping gene HPRT. $N_{+/+}$=3; $N_{ob/ob}$=3; n.s.: not significant, two-tailed t-test. Data are shown as mean ± SEM, source data are provided as a Source Data file.

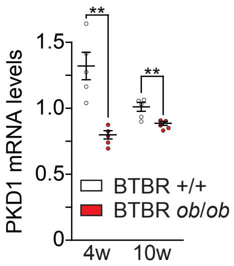

Supplementary Figure 10. Analysis of PKD1 (Prkcm) expression in islets of wt and *ob*/*ob* BTBR mice (from Keller et al., Genome Res. 2008). RNA seq using mRNA isolated from pancreatic islets of 4-week old and 10-week old BTBR +/+ and BTBR *ob*/*ob* mice.The expression values were normalized to the reference pool derived from all 20 individuals (lean and *ob*/*ob* at 4 and 10 weeks of age, n=5 for each group). $N_{4w,\ +/+}$=5; $N_{4w,\ ob/ob}$=5; $N_{10w,\ +/+}$=5; $N_{10w,\ ob/ob}$=5. **P<0.01, two-tailed t-test. Lines represent mean ± SEM, source data are provided as a Source Data file.

a

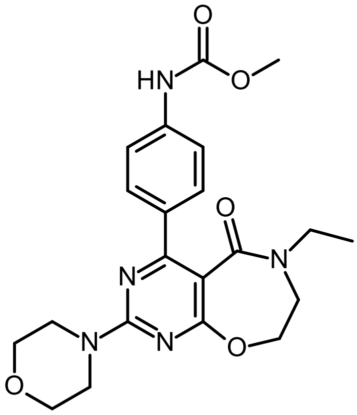

Compound A - MW: 427.45 ($C_{21}H_{25}N_5O_5$)

b

	**p38δ** IC50 (nM)	**p38α** IC50 (nM)	**p38γ** IC50 (nM)	**PKD1** IC50 (nM)
**Compound A**	380	> 30 μM	710	> 30 μM

c

Kinases	Compound A 1 μM	Compound A 10 μM
Abl(h)	6	8
ALK(h)	4	0
AMPKα2(h)	-5	5
Aurora-B(h)	-24	-28
BrSK2(h)	5	5
BTK(h)	-1	0
CaMKI(h)	5	11
CaMKIIδ(h)	-6	1
CDK1/cyclinB(h)	-5	4
CDK5/p25(h)	-2	-2
CDK9/cyclin T1(h)	-16	3
CLK2(h)	-2	-2
cKit(h)	4	6
c-RAF(h)	-5	2
cSRC(h)	-9	-12
DDR2(h)	6	15
DYRK2(h)	-10	-10
EGFR(h)	-17	4
EphA1(h)	0	7
EphA3(h)	8	12
EphB1(h)	6	10
EphB4(h)	-3	8
FGFR1(h)	9	6
Flt1(h)	-30	-28
Flt3(h)	-30	-25
GSK3α(h)	-9	-7
GSK3β(h)	1	12
Hck(h) activated	-5	5
IKKε(h)	16	18
IR(h)	-21	-14

Kinases	Compound A 1 μM	Compound A 10 μM
JAK3(h)	0	-5
KDR(h)	-16	-12
Lck(h) activated	-7	13
Lyn(h)	-2	13
LRRK2(h)	-18	-11
MAPK1(h)	0	5
MELK(h)	2	9
Met(h)	-16	-7
MST2(h)	-16	-2
mTOR(h)	-1	48
p70S6K(h)	15	59
PAK4(h)	-2	4
PEK(h)	-12	-11
PDGFRα(h)	1	0
Pim-1(h)	-6	-7
Pim-2(h)	8	7
PKA(h)	-8	-22
PKBα(h)	14	17
PKCβII(h)	0	1
PKCε(h)	8	12
PKCθ(h)	7	7
PKCζ(h)	0	10
Plk1(h)	2	11
Ron(h)	10	9
Rsk1(h)	-11	5
SAPK2a(h)	-16	9
SAPK3(h)	11	64
Syk(h)	4	20
TrkA(h)	-21	5
PI3 Kinase (p110α/p85α)(h)	-1	2

>90% Inhibition
75-90% Inhibition
50-75% Inhibition
35-50% inhibition
<35% no change

d

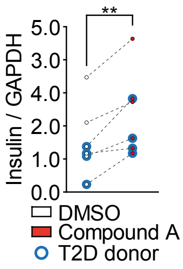

Supplementary Figure 11. Generation of Compound A selectively inhibiting p38δ. (**a**) Chemical structure of Compound A. (**b**) Selectivity profile of Compound A versus p38δ, p38α, p38γ and PKD1. (**c**) Eurofins Pharma Discovery Services Kinase ProfilerTM (60 kinases). (**d**) Quantification of immunoblots shown in **Fig. 4g** and **h**. Batches of islets were independently isolated from 2 non-diabetic and 4 T2D donors and treated as indicated. **P<0.01, two-tailed, paired t-test. Source data are provided as a Source Data file.

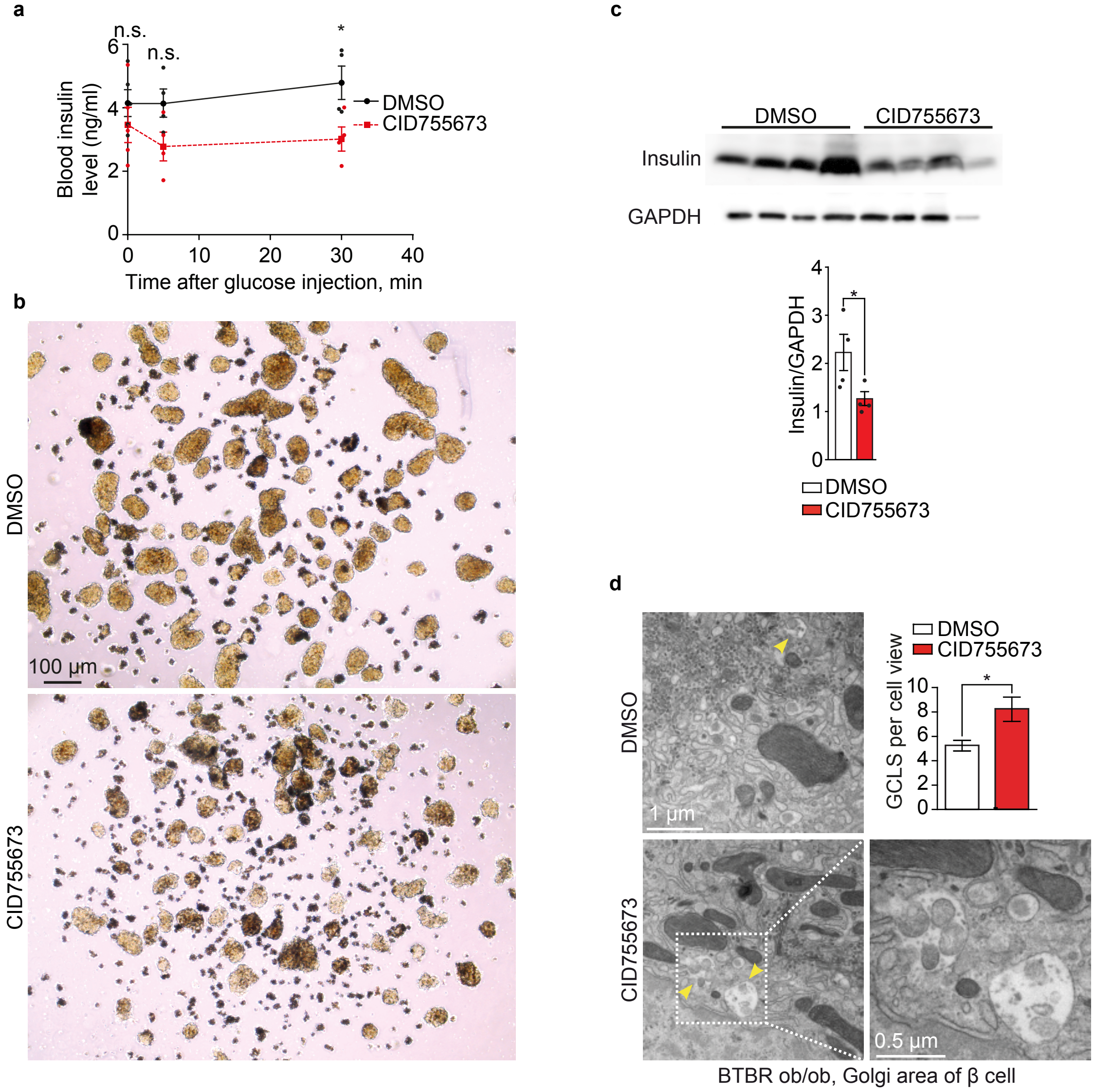

Supplementary Figure 12. PKD Inhibition *in vivo* reduces insulin secretion and insulin content levels, and increases the number of granule-containing lysosomes (GCLs) in pancreatic β cells. (**a**) Blood insulin levels in response to glucose injection in BTBR ob/ob mice implanted with osmotic pumps containing control solvent (DMSO) or CID755673. N_{DMSO}=5; $N_{CID755673}$=5. *P<0.05, two-tailed t-test. (**b**) Brightfield image of freshly isolated islets derived from BTBR ob/ob mice, treated with DMSO or CID755673. (**c**) Immunoblot of insulin using lysates of islets isolated from BTBR ob/ob mice, treated with DMSO or CID755673. GAPDH was used as a loading control. Quantification of Insulin/GAPDH ratio. N=4 per group; *P<0.05, two-tailed t-test. (**d**) Electron microscopy of β cells of islets isolated from BTBR ob/ob mice implanted with osmotic pumps containing control solvent (DMSO) or CID755673. Arrowheads point to GCLs. Quantification of GCLs per cell view. N_{DMSO}=25 cells and $N_{CID755673}$=27 cells from 5 islets per each group. *P<0.05, Mann-Whitney U-test. In **a**, **c** and **d** data are shown as mean ± SEM, source data are provided as a Source Data file.

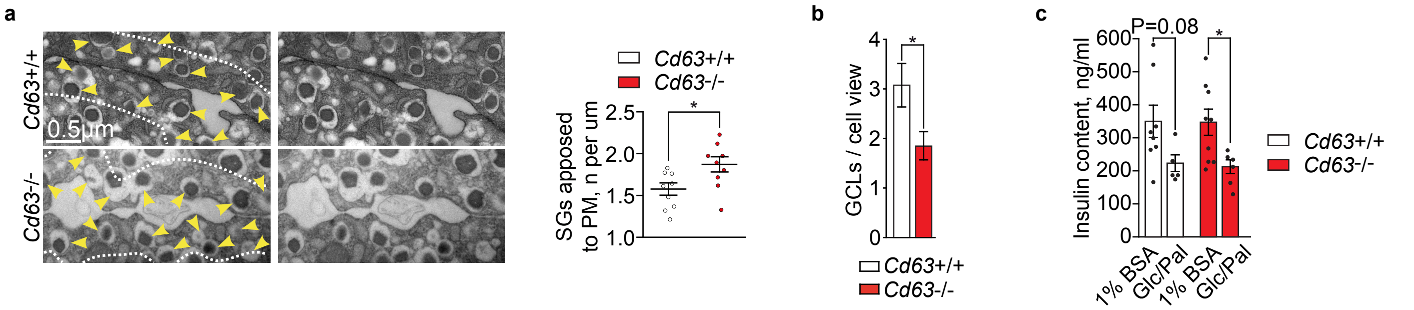

Supplementary Figure 13. Increased amount of secretory granules (SGs) in plasma membrane (PM) area and decreased amount of GCLs in Golgi area of β cells of *Cd63-/-* mice. (**a**) Electron microscopy of β cells of islets isolated from *Cd63*+/+ and *Cd63*-/- mice. Arrowheads point to SGs in PM area. PM length (uM) and SGs in the PM area (defined as a band of 500 nm thickness under PM) were quantified in 9 fields of view from 3 mice per each genotype. Quantification of SGs per PM, um. *P<0.05, two-tailed t-test. Lines represent mean ± SEM. (**b**) Quantification of granule-containing lysosomes (GCLs) per cell view. *Cd63*+/+ group is identical to shown in **Supplementary Figure 7c** (chow diet). $N_{Cd63+/+}$=27; $N_{Cd63-/-}$=58 from 3 mice per group; *P<0.05, two-tailed t-test. (**c**) Total insulin content levels from experiment shown in **Figure 5f**. Batches of islets were independently isolated from *Cd63*+/+ and *Cd63*-/- mice; $N_{1\%\ BSA}$ =8 (*Cd63*+/+), 9 (*Cd63*-/-), $N_{Glc/Pal}$= 5 (*Cd63*+/+), 6 (*Cd63*-/-), *P<0.05, two-tailed t-test. Data are shown as mean ± SEM, source data are provided as a Source Data file.

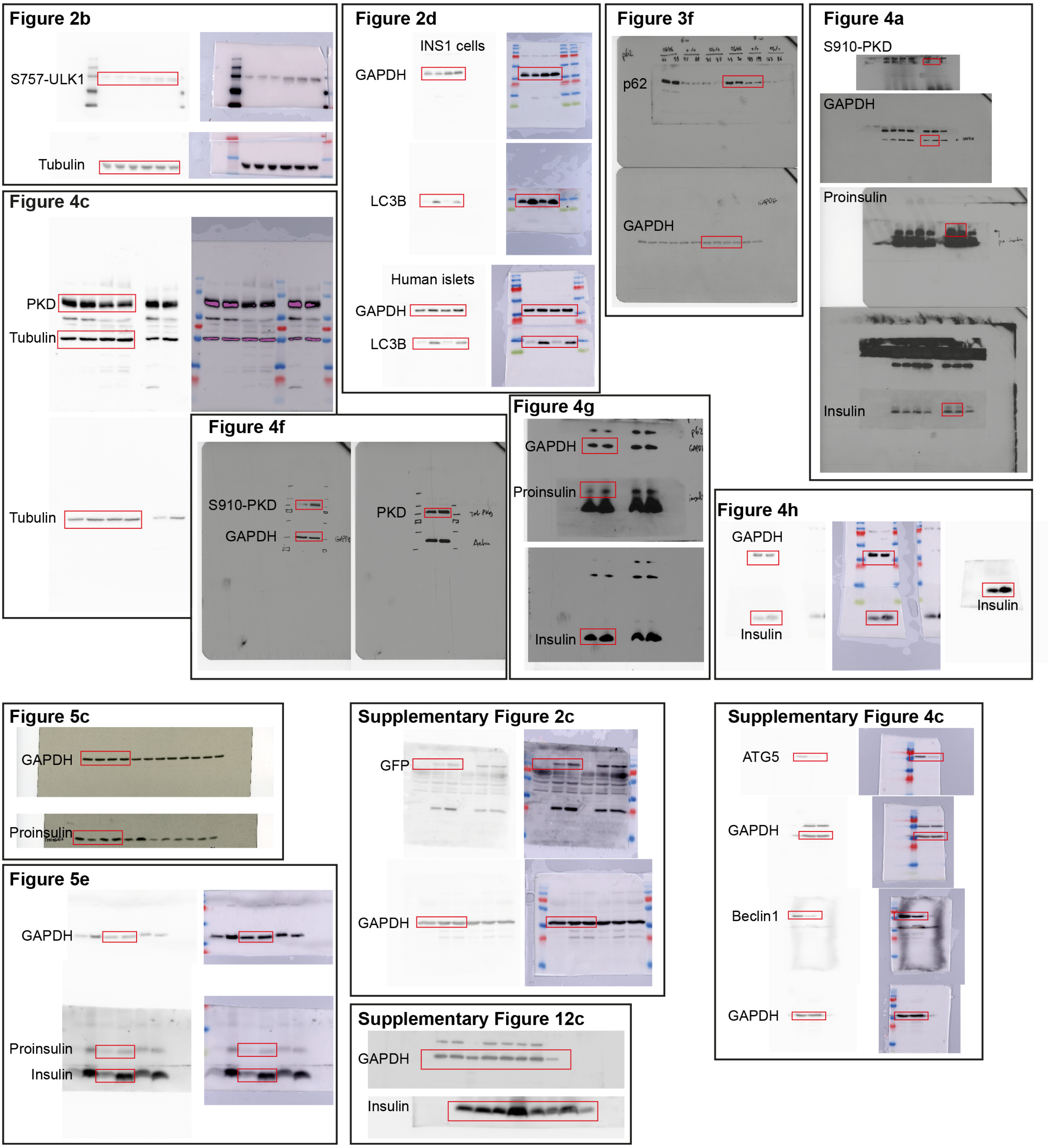

Supplementary Figure 14. Uncropped scans of immunoblots presented in indicated figures.
