# Supplementary material for: Lysosomal degradation of newly formed insulin granules contributes to β cell failure in diabetes
Source: Nat Commun. 2019 Jul 25;10:3312. doi: 10.1038/s41467-019-11170-4 (PMC6658524; doi:10.1038/s41467-019-11170-4)
Supplement: Supplementary file 2 — Description of Additional Supplementary Files [file 41467_2019_11170_MOESM2_ESM.pdf]

Description of Additional Supplementary Files

File name: Supplementary Movie 1

Description: Live-cell imaging of $INS1^{PGCD}$ cells endogenously expressing Phogrin-GFP (Phogrin-GFP^{endo}, green) and CD63-DsRed (CD63-$DsRed^{endo}$, red), pre-treated with Glc/Pal for 24h (shown in Supplementary Fig. 5b).

File name: Supplementary Movie 2

Description: Related to Fig. 2a: immunofluorescence of CD63 (green) and mTOR (red) in INS1 cells treated with glucolipotoxic (1% BSA, 33.3 mM Glucose and 0.4 mM Palmitate) media for 20h. Golgi-CFP was used to visualize the Golgi apparatus.

File name: Supplementary Movie 3

Description: Related to Fig. 3c, d: 3D reconstruction of the region of β cell with the granule-containing lysosomes (yellow), secretory granules (green) and lysosomes (red) revealed by Focused Ion Beam Scanning Electron Microscopy (FIB-SEM) of 6-week old BTBR ob/ob mice.
