# Supplementary material for: Lysosomal degradation of newly formed insulin granules contributes to β cell failure in diabetes
Source: Nat Commun. 2019 Jul 25;10:3312. doi: 10.1038/s41467-019-11170-4 (PMC6658524; doi:10.1038/s41467-019-11170-4)
Supplement: Supplementary file 6 — Reporting Summary [file 41467_2019_11170_MOESM6_ESM.pdf]

nature research

Corresponding author(s): Romeo Ricci, Alexander Goginashvili

Last updated by author(s): May 27, 2019

Reporting Summary

Nature Research wishes to improve the reproducibility of the work that we publish. This form provides structure for consistency and transparency in reporting. For further information on Nature Research policies, see Authors & Referees and the Editorial Policy Checklist.

Statistics

For all statistical analyses, confirm that the following items are present in the figure legend, table legend, main text, or Methods section.

n/a	Confirmed	
☐	☒	The exact sample size (*n*) for each experimental group/condition, given as a discrete number and unit of measurement
☐	☒	A statement on whether measurements were taken from distinct samples or whether the same sample was measured repeatedly
☐	☒	The statistical test(s) used AND whether they are one- or two-sided<br>*Only common tests should be described solely by name; describe more complex techniques in the Methods section.*
☒	☐	A description of all covariates tested
☒	☐	A description of any assumptions or corrections, such as tests of normality and adjustment for multiple comparisons
☐	☒	A full description of the statistical parameters including central tendency (e.g. means) or other basic estimates (e.g. regression coefficient) AND variation (e.g. standard deviation) or associated estimates of uncertainty (e.g. confidence intervals)
☐	☒	For null hypothesis testing, the test statistic (e.g. *F*, *t*, *r*) with confidence intervals, effect sizes, degrees of freedom and *P* value noted<br>*Give P values as exact values whenever suitable.*
☒	☐	For Bayesian analysis, information on the choice of priors and Markov chain Monte Carlo settings
☒	☐	For hierarchical and complex designs, identification of the appropriate level for tests and full reporting of outcomes
☒	☐	Estimates of effect sizes (e.g. Cohen's *d*, Pearson's *r*), indicating how they were calculated

Our web collection on statistics for biologists contains articles on many of the points above.

Software and code

Policy information about availability of computer code

Data collection	No software was used for data collection
Data analysis	Open source FIJI plugins have been used

For manuscripts utilizing custom algorithms or software that are central to the research but not yet described in published literature, software must be made available to editors/reviewers. We strongly encourage code deposition in a community repository (e.g. GitHub). See the Nature Research guidelines for submitting code & software for further information.

Data

Policy information about availability of data

All manuscripts must include a data availability statement. This statement should provide the following information, where applicable:

- Accession codes, unique identifiers, or web links for publicly available datasets
- A list of figures that have associated raw data
- A description of any restrictions on data availability

All relevant data that support the conclusions of this study are available from the authors on reasonable request (see author contributions for specific data sets). The source data underlying all quantitative analyses presented in this study are provided as a Source Data file.

Field-specific reporting

Please select the one below that is the best fit for your research. If you are not sure, read the appropriate sections before making your selection.

☒ Life sciences ☐ Behavioural & social sciences ☐ Ecological, evolutionary & environmental sciences

Life sciences study design

All studies must disclose on these points even when the disclosure is negative.

Sample size	Sample size estimate has been performed based on previous experience to obtain statistical significance and reproducibility.
Data exclusions	Only exclusions have been made in case of failed experimental procedures (based on pre-established criteria).
Replication	All experiments underlying main conclusions of this study have been successfully replicated multiple times and corroborated by several models.
Randomization	Samples and organisms were randomly allocated to experimental groups. No specific randomization protocol has been used. Mice were age-matched and sex-matched (littermates whenever possible).
Blinding	No specific blinding was applied. In addtion, for the mice experiments, control and experimental groups progressively developed easily distinguishable diabetes related phenotypes (compound vs control treatments etc.), that served as an additional corroboration of study's conclusions (but made blinding unrealistic).

Reporting for specific materials, systems and methods

We require information from authors about some types of materials, experimental systems and methods used in many studies. Here, indicate whether each material, system or method listed is relevant to your study. If you are not sure if a list item applies to your research, read the appropriate section before selecting a response.

Materials & experimental systems

n/a	Involved in the study	
☐	☒	Antibodies
☐	☒	Eukaryotic cell lines
☒	☐	Palaeontology
☐	☒	Animals and other organisms
☒	☐	Human research participants
☒	☐	Clinical data

Methods

n/a	Involved in the study	
☒	☐	ChIP-seq
☒	☐	Flow cytometry
☒	☐	MRI-based neuroimaging

Antibodies

Antibodies used

Information on antibodies used and specific conditions is provided in Methods/Primary antibodies used:
Fig. 1a:
Insulin, Cell Signaling Technology (CST), produced in rabbit (4590) (1/100)
Giantin G1/133, Enzo life science monoclonal, mouse, ALX-804-600-C100) (1/1000)
Fig. 1b:
Insulin, CST, produced in rabbit (4590) (1/100)
Anti rat-CD63 produced in mouse Biorad (AD-1), formerly Serotec (1/100)
Fig. 2a and Supplementary Video 2:
Anti rat-CD63 produced in mouse Biorad (AD-1), formerly Serotec (1/100)
mTOR, 7C10, CST, produced in rabbit (2983) (1/200)
Fig. 2b:
Phospho-ULK1 (Ser757), CST produced in rabbit (6888)
Fig. 2d:
LC3B antibodies: (1) 2G6, produced in mouse, Nanotools (1/1000) and (2) Novus, produced in rabbit, (NB100-2220) (1/1000)
GAPDH, Sigma, produced in rabbit (G9545) (1/10000)
Fig. 3a:
Insulin, CST, produced in mouse L6B10 (8138) (1/100)
CD63, produced in rabbit, from P. Saftig (1/100)
Fig. 3e:
Insulin, CST, produced in rabbit 4590 (1/100)
p62, Progen, produced in guinea pig (GP62-C) (1/100)
Fig. 3f:
p62, Progen, produced in guinea pig (GP62-C) (1/1000)
GAPDH Sigma produced in rabbit (G9545) (1/10 000)
Fig. 4a:
Anti-phospho-PRKD1 (pSer910), Sigma, produced in rabbit (SAB4300075) (1/1000)
Tubulin, Sigma, produced in mouse (T9026) (1/10000)
Insulin, Sigma, produced in guinea Pig (I8510)
Fig. 4c:
PKD/PKCµ Antibody, CST, rabbit (2052) (1/1000)

Tubulin, Sigma, produced in mouse (T9026) (1/10000)
Fig. 4f:
Anti-phospho-PRKD1 (pSer910), Sigma, produced in rabbit (SAB4300075) (1/1000)
PKD/PKCμ Antibody, CST, rabbit (2052) (1/1000)
GAPDH Sigma produced in Rabbit (G9545) (1/10000)
Fig. 4g and h:
Insulin, Sigma, produced in Guinea Pig (I8510)
GAPDH Sigma produced in Rabbit (G9545) (1/10000)
Fig. 5a:
Insulin, CST, produced in Rabbit (4590) (1/100)
Fig. 5b:
Insulin, Sigma, produced in mouse K36AC10 (I2018) (1/1000)
Fig. 5c:
C-peptide I, produced in mouse, Biorad, Formerly Serotec (MCA2857) (1/1000)
GAPDH Sigma produced in Rabbit (G9545) (1/10000)
Fig. 5d:
Insulin, CST, produced in Rabbit (4590) (1/100)
Anti rat-CD63 produced in Mouse Biorad (AD-1), formerly Serotec (1/100)
Fig. 5e:
Insulin, Sigma, produced in Guinea Pig (I8510)
GAPDH Sigma produced in Rabbit (G9545) (1/10000)
Supplementary Fig. 1a:
Insulin, Sigma, produced in mouse K36AC10 (I2018) (1/1000)
Insulin, CST, produced in Rabbit (4590) (1/100)
Supplementary Fig. 1b:
Insulin, CST, produced in Mouse L6B10 (8138) (1/100)
Lamp2, Invitrogen, produced in Rabbit (1/700)
Supplementary Fig. 2c:
GFP, ThermoFisher Scientific, produced in rabbit, A6455
GAPDH, Sigma, produced in Rabbit (G9545) (1/10000)
Supplementary Fig. 2e:
Insulin, CST, produced in Rabbit (4590) (1/100)
Supplementary Fig. 2i:
Anti rat-CD63 produced in Mouse Biorad (AD-1), formerly Serotec (1/100)
Supplementary Fig. 4a:
Insulin, CST, produced in Rabbit (4590) (1/100)
Supplementary Fig. 4b:
An antibody against Phogrin was a generous gift from John Hutton and Howard Davidson (University of Colorado) (1/1000)
Supplementary Fig. 4c:
Beclin-1, CST, produced in rabbit (3495) (1/1000)
ATG5, CST, produced in rabbit (12994) (1/1000)
GAPDH Sigma produced in Rabbit (G9545) (1/10000)
Supplementary Fig. 4i:
LC3B antibody (PM036) from MBL
Supplementary Fig. 7d:
Insulin, CST, produced in Mouse L6B10 (8138) (1/100)
CD63, produced in Rabbit, from P. Saftig (1/100)

Validation

We have successfully validated all the antibodies used in the study (for specific applications: immunoblotting, immunofluorescence etc.) using knockdown/knockout, co-localization or positive control (for ELISA) approaches.

Eukaryotic cell lines

Policy information about cell lines

Cell line source(s)

INS1 cells were kindly provided by Prof. Wollheim (University of Geneva, Switzerland).

Authentication

This celll line is routinely used in research field (including 10+ years for our lab) and has been thoroughly tested by us and others. Tests included sequence verification, secretion assays, expression of various markers etc.

Mycoplasma contamination

All cell lines were tested negative for mycoplasma.

Commonly misidentified lines
(See ICLAC register)

none of commonly misidentified cell line has been used

Animals and other organisms

Policy information about studies involving animals; ARRIVE guidelines recommended for reporting animal research

Laboratory animals

BTBR ob/+ mice were obtained from Charles River and were maintained by heterozygous breeding to generate +/+ and ob/ob littermates. CD63-/- mice were generated and described previously . Mice were housed under controlled temperature on a 12-h light/dark cycle with unrestricted access to water and standard laboratory chow. Male mice were used in all experiments.

Wild animals	the study did not involved wild animals
Field-collected samples	the study did not involved field-collected samples
Ethics oversight	Maintenance and animal experimentation were in accordance with the local ethical committee (Com'Eth) in compliance with the European legislation on care and use of laboratory animals.

Note that full information on the approval of the study protocol must also be provided in the manuscript.
